# Sunscreen Effect Exerted by Secondary Carotenoids and Mycosporine-like Amino Acids in the Aeroterrestrial Chlorophyte *Coelastrella rubescens* under High Light and UV-A Irradiation

**DOI:** 10.3390/plants10122601

**Published:** 2021-11-26

**Authors:** Anna Zaytseva, Konstantin Chekanov, Petr Zaytsev, Daria Bakhareva, Olga Gorelova, Dmitry Kochkin, Elena Lobakova

**Affiliations:** 1 Department of Bioengineering, Faculty of Biology, Lomonosov Moscow State University, 1-12 Leninskie Gory, 119192 Moscow, Russia; kublanovskaya@mail.bio.msu.ru (A.Z.); zaitsev@mail.bio.msu.ru (P.Z.); snow.winter99909@gmail.com (D.B.); ogo439@mail.ru (O.G.); elena.lobakova@gmail.com (E.L.); 2 Centre for Humanities Research and Technology, National Research Nuclear University MEPhI, 31 Kashirskoye Highway, 115522 Moscow, Russia; 3 N.N. Semyonov Federal Research Center for Chemical Physics, Russian Academy of Science, 4 Kosygina Street, Building 1, 119192 Moscow, Russia; 4 Department of Plant Physiology, Faculty of Biology, Lomonosov Moscow State University, 1-12 Leninskie Gory, 119192 Moscow, Russia; dmitry-kochkin@mail.ru; 5 Timiryazev Institute of Plant Physiology, Russian Academy of Science, Botanicheskaya Street 35, 127276 Moscow, Russia

**Keywords:** photoprotection, carotenoids, mycosporine-like amino acids, sunscreens, autophagy, aeroterrestrial algae

## Abstract

The microalga *Coelastrella rubescens* dwells in habitats with excessive solar irradiation; consequently, it must accumulate diverse compounds to protect itself. We characterized the array of photoprotective compounds in *C. rubescens*. Toward this goal, we exposed the cells to high fluxes of visible light and UV-A and analyzed the ability of hydrophilic and hydrophobic extracts from the cells to absorb radiation. Potential light-screening compounds were profiled by thin layer chromatography and UPLC-MS. *Coelastrella* accumulated diverse carotenoids that absorbed visible light in the blue–green part of the spectrum and mycosporine-like amino acids (MAA) that absorbed the UV-A. It is the first report on the occurrence of MAA in *Coelastrella*. Two new MAA, named coelastrin A and coelastrin B, were identified. Transmission electron microscopy revealed the development of hydrophobic subcompartments under the high light and UV-A exposition. We also evaluate and discuss sporopollenin-like compounds in the cell wall and autophagy-like processes as the possible reason for the decrease in sunlight absorption by cells, in addition to inducible sunscreen accumulation. The results suggested that *C. rubescens* NAMSU R1 accumulates a broad range of valuable photoprotective compounds in response to UV-A and visible light irradiation, which indicates this strain as a potential producer for biotechnology.

## 1. Introduction

The surfaces of stones, buildings and tree bark can be covered by an orange or red crust. This crust is of a biological nature: it is formed by aeroterrestrial microalgae. These algae are among the most ubiquitous microbes on the aerial surface [1]. Aeroterrestrial microalgae exist under harsher and more variable environmental conditions than freshwater, marine, and soil species; therefore, they have to exhibit effective protective mechanisms against adverse abiotic factors [2,3].

Microalgae are oxygenic phototrophic organisms. On the one hand, light is vitally important for their existence, because it promotes electron transport in the photosynthetic electron transport chain (ETC), a main source of assimilatory power for the synthesis of organic substances in the cell. On the other hand, it is the cause of photodamage, a set of processes leading to light-driven destructions in the cell. Thus, phototrophic organisms must constantly avoid and dispose of the harmful power of electromagnetic radiation. Being exposed to the sunlight directly falling on the surface of the earth, aeroterrestrial species are especially vulnerable to photodamage [2,3,4]. In the process of evolution, they have acquired effective photoprotective mechanisms, including the synthesis of screening compounds that effectively absorb solar radiation (sunscreens). The repertoire of sunscreen substances generated by the cell must reflect the spectral composition of the absorbed light [1,2,3,5]. Although there are annual, seasonal and diurnal changes in sunlight spectral quality, strongly depending on geographical region [6,7,8], some common trends in its spectral features are detectable. Sunlight that directly falls on the earth surface contains the whole spectrum of photosynthetic radiation (PAR), in the range of 400-700 nm, as well as UV radiation.

UV radiation, in accordance with CIE (Commission Internationale de l’Eclairage, International Commission on Illumination), is divided into UV-C (190–280 nm) UV-B (280–315 nm) and UV-A (315–400 nm). A significant part of UV-B is absorbed by the stratospheric ozone layer. Therefore, the light near the surface of the earth is enriched by UV-A [9]. UV radiation is well-known for its destructive effect on living organisms [10,11,12]. It is absorbed by aromatic rings of nucleotides and amino acids that cause DNA and protein damage, respectively [9,13]. Disulfide bonds of proteins are also a target of UV [9,13]. Besides that, UV promotes photochemical reactions of reactive oxygen species (ROS) formation. It results in photo-oxidative damage, especially in the cell membranes, where free radical chain reactions involving ROS and unsaturated fatty acid residues occur [9,11,13]. Additionally, in photosynthetic oxygenic organisms, UV-A induces the degradation of catalytic Mn-cluster of water-oxidizing complexes, D1 and D2 proteins of the PS II RC, as well as PS II Q_A_- and Q_B_-binding sites [10]. Following UV-screening compounds could be listed. Many terrestrial cyanobacteria accumulate the pigment scytonemin, a product of the condensation of tryptophan and phenylpropanoid subunits, with the absorption maximum at 370 nm [12,13]. Mycosporines and mycosporine-like amino acids (MAA) are representatives of a diverse group of UV-protecting compounds [2,4,9,12,13,14,15,16,17,18,19,20,21,22,23,24]. They are produced by fungi, bacteria (including Cyanobacteria) and eukaryotic microalgae. MAA have been also found in animal organisms (most likely due to their diet or the horizontal transfer of the genes of their synthesis) [4,11,12,13,14,15,16,17,18,19,20,21,22,23]. They exhibit a maximum in the UV-A region due to cyclohexenone or cyclohexenimine structural elements in their molecules [4,9,12,14,15,16,17,18]. Phenylpropanoids including flavones, flavanols, cinnamoyl esters, and anthocyanins are typical sunscreens of higher plants [10,12,13]. Streptophytes accumulates phenolic compounds [3,9,12,13,25]. Polyphenolics were suggested to occur also in the cell wall of the cyst stages of the snow alga *Chloromonas krienitzii* [26]. Animals and fungi accumulate melanins, highly stable polymeric substances, effectively absorbing radiation from visible and UV regions [12]. Carotenoids are accumulated as protective substances in a response to UV radiation in several species of higher plants and microalgae [10], particularly in aeroterrestrial species [2,27]. Many UV-protective metabolites, such as usnic acid, atranorin, nephrarctin, and phenarctin, have been found in lichens [28,29,30]. The lichen *Xanthoria parietina* synthesizes parietin [31]. Screening compounds are produced by mycobiont and protect lichen photobionts [28].

Under suboptimal conditions, absorbance of PAR by photosynthetic pigments increases the risk of photodamage, mainly due to ROS production [9,32]. There are following main pigments shielding photosynthetic apparatus (PSA) against visible light. Particularly, plants produce anthocyanins [33,34,35] and betalains [27,34]. In *Aloe*, *Cryptomeria*, *Metasequoia*, *Taxodium*, *Chamaecyparis*, *Buxus*, and *Thuja* [34,36,37], screening is realized through ketocarotenoids. In the cells of some green microalgae (Chlorophyceae), carotenoids also play a role of PAR absorbing sunscreens. Particularly, the microalga *Haematococcus lacustris* (Chlorophyceae, Volvocales) accumulates high amounts of the ketacarotenoid astaxanthin shown to have a photoprotective effect as a shielding agent [37,38,39,40,41,42,43,44,45]. Another microalga, *Dunaliella salina* (Volvocales), accumulates β-carotene that protects its cells against damage from high irradiation by screening through absorption in the blue region of the spectrum [42,46,47]. Such carotenoids are usually produced in large quantities under stressed conditions, e.g., nitrogen source deficiency and high light intensity, and located independently on PSA [48]. Members of some other genera of green microalgae have also been reported as carotenoid accumulating, e.g., *Bracteacoccus* [49,50,51], *Chloromonas* [26], *Sanguina* [52], *Chromochloris* [44,53], and *Coelastrella* [42,51,54,55,56,57,58,59,60].

*Coelastella rubescens* (Chlorophyceae, Scenedesmaceae) (Vinatzer) Kaufnerová & Eliás is a typical aeroterrestrial microalga [57]. It forms a dry crust on the soil, on either human-made or natural surfaces. We selected the strain defined as *C. rubescens* NAMSU R1, isolated from the crust on the tree bark, as a typical representative of aeroterrestrial photoautotrophs. We attempted to draw a full picture of the sunscreen effects in it, and summarized the results based on the previous data and our findings. Toward this end, we subjected *C. rubescens* to UV-A and excessive PAR and revealed the repertoire of its possible photoprotection compounds providing the vitally important ability to screen the sunlight, enabling the algae to dwell in the aeroterrestrial environment.

## 2. Results and Discussion

### 2.1. Identification and Characterization of a New Strain Coelastrella Rubescens NAMSU R1

**Figure 1:**
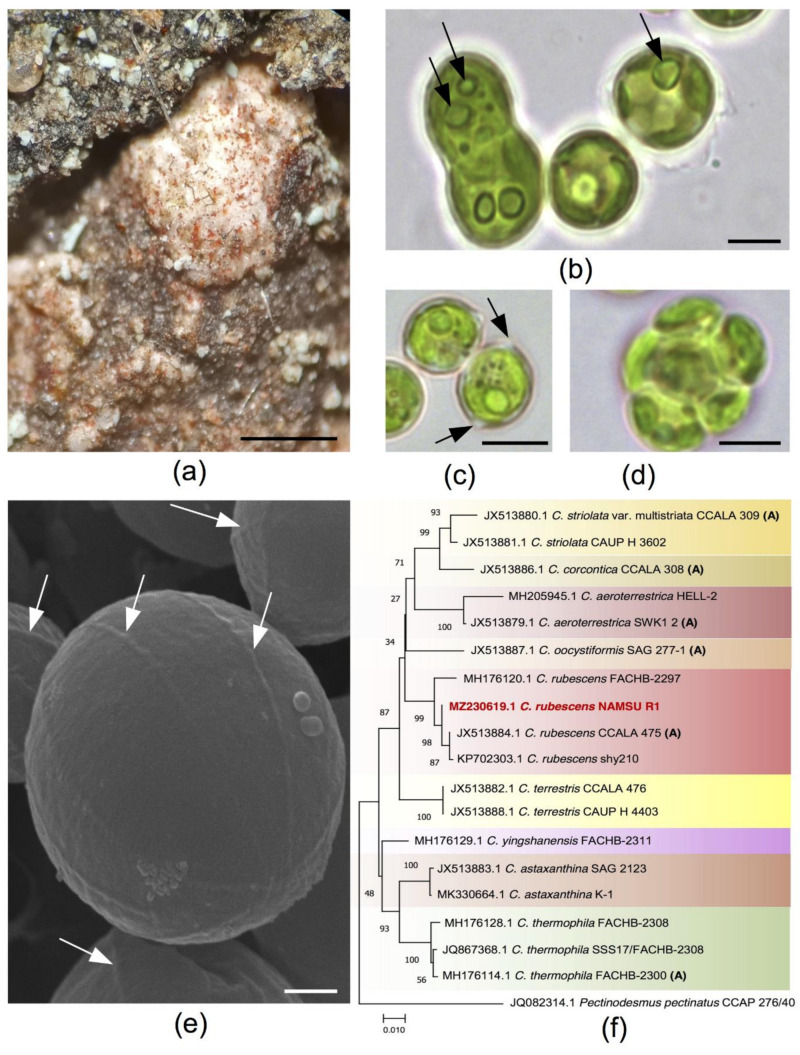
The characterization of a new strain NAMSU R1: (**a**) algal colonies in the natural habitat represented by a reddish crust on the surface of the tree bark; (**b**) features of the vegetative cells with one or two pyrenoids (arrows); (**c**) cells with polar thickenings (arrows); (**d**) aplanosporangium with aplanospores; (**e**) a surface of the cells investigated by scanning electron microscopy, meridional ribs are pointed out by arrows; (**f**) phylogenetic tree of the strain NAMSU R1, based on the ITS1-5.8S rRNA-ITS2 fragment inferred by the Maximum Likelihood (ML) algorithm. The percentages of the bootstrap support are shown near corresponding nodes; names of species and strain as well as GenBank accession numbers are shown for taxa; authentic strains are marked by ‘A’; studied strain is marked by red; tree is drawn to scale in the units of substitution number per the total number of positions in the multiple alignment. Scale bars: 500 μM (**a**), 5 μM (**b**–**d**), 1 μM (**e**).

The strain NAMSU R1 was obtained from the surface of the bark from an apple tree (*Malus × domestica)* in Rastorguevo Village, Moskovskaya oblast, Russia (Figure 1a). After a series of reseedings on the medium, it was represented by monoalgal cultures of green-colored spherical cells of 5–10 μM in diameter, as well as dividing cells with parietal chloroplast containing a pyrenoid (Figure 1b). In some cells, two pyrenoids were observed (Figure 1b), which is unusual for *Coelastrella* species [54,55]. In some cases, cells formed clusters. Additionally, cells with two polar thickenings of the cell wall (Figure 1c)—common for algae from Scenedesmaceae, including *Coelastrella* [57]—were observed in the young culture in the liquid medium. Sporangia with 2–8 aplanospores were present. All these results were in accordance with the literature data on *Coelastrella* [55] (Figure 1d).

SEM observations revealed the presence of prominent meridional ribs on the surface (Figure 1e), which is typical for the *Coelastrella* genus of green algae (Sphaeropleales, Scenedesmaceae) [57,58,59]. According to these and light microscopy data, the microalga was primarily identified as *Coelastrella* sp. NAMSU R1. Based on the analysis of the fragment of the nuclear ribosomal gene cluster, including internal transcribed spacers (internal transcribed spacer, ITS) 1 and 2, as well as the 5.8S rRNA gene (ITS1-5.8S rRNA-ITS2), it clustered with representatives of the species *C. rubescens* (Vinatzer) Kaufnerová and Eliás, including the type of strain (Figure 1f). Thus, the strain was defined as *C. rubescens* NAMSU R1. This species is a typical example of aeroterrestrial microalgae inhabiting terrestrial surfaces with a different humidity [58]. The sequence of the ITS1-5.8S rRNA-ITS2 fragment was submitted to the NCBI GenBank database under the accession number MZ230619.1.

### 2.2. Sunscreen Effect in the Visible Region of the Spectrum

In the current work, we have followed an effect of sunscreen compounds in the response to UV-A and PAR, which are the main components of solar radiation near the surface of the earth. To reveal this effect in the aeroterrestrial chlorophyte *C. rubescens*, we followed the main criteria of sunscreen compounds proposed by Cockell and Knowland [12]. The compound should absorb the light from the range of PAR and/or UV; accumulation of such compound should be radiation-inducible, and the sunscreen effect should be demonstrated in vivo [12]. Moreover, the accumulation of sunscreens should prevent the possible negative effects of photodamage, such as photoinhibition [12].

**Figure 2:**
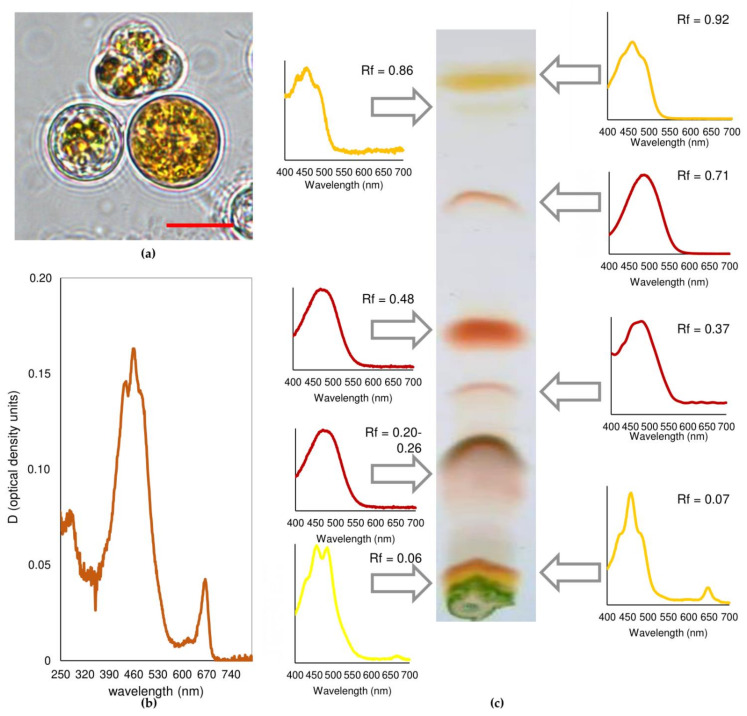
Carotenoid accumulation in the cells of *Coelastrella rubescens* NAMSU R1: (**a**) typical orange-colored cells after HL+UV-A treatment, scale bar: 7 μm; (**b**) representative absorbance spectrum of the chloroform extract of *C. rubescens* NAMSU R1 after HL+UV-A treatment; (**c**) separation of the chloroform extract of *C. rubescens* NAMSU R1 cells after HL+UV-A treatment by thin layer chromatography, absorbance spectra in the acetone and R*_f_* are shown for pigment fractions.

**Figure 3:**
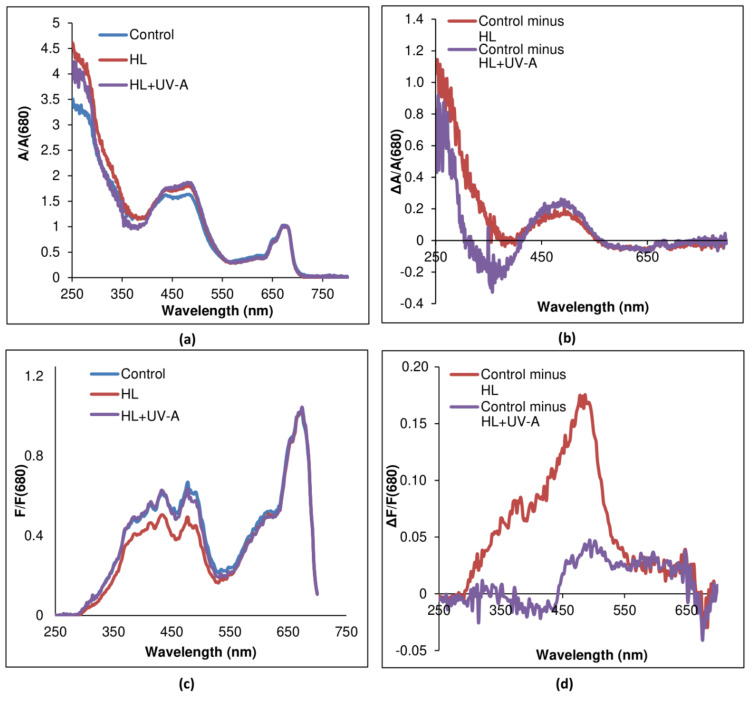
Spectral characteristics of *Coelastrella rubescens* NAMSU R1 cells and their water–methanol extracts: (**a**) absorbance spectra of cell suspensions compensated to light scattering normalized to the red maximum; (**b**) differential absorbance spectra of the cells compensated to light scattering; (**c**) excitation spectra of the cells normalized to the red maximum (detection at 750 nm); (**d**) differential excitation spectra of the cells. HL—cells and extracts of the cells after HL treatment. HL+UV-A—cells and extracts of the cells after HL and UV-A treatment. Control—cells treated by LL.

After 21 days of cultivation under high light (HL) or HL and UV-A (HL+UV-A), the *C. rubscens* NAMSU R1 cells became orange (Figure 2a). Based on the shape of the absorbance spectra of *C. rubescens* NAMSU R1 chloroform extracts, a peak in the range of 480–485 nm characterizing ketocarotenoids was observed, as well as a peak in the UV region (about 280 nm) (Figure 2b). The profile of carotenoids was determined by their separation by thin layer chromatography (Figure 2c). The absorbance spectra of the *C. rubescens* NAMSU R1 suspensions compensated to light scattering were also characterized by increasing the absorbance in the blue–green region (Figure 3a).

Analysis of differential spectra (exposed to HL or HL+UV-A minus control) revealed the presence of peaks with the maximum at c.a. 495 nm (Figure 3b). It was a sign of the accumulation of ketocarotenoids in the cell. In terms of carotenoid amount, their content increased from 0.4 ± 0.1% to 2.8 ± 0.3%, and to 2.3 ± 0.1% of cell dry mass during HL and HL+UV-A treatment, respectively. Ketocarotenoid accumulation is a typical response of some green algae to high levels of irradiation and some other stress factors, e.g., nutrient deficiency and ROS generation [37,38,44,45]. PSA is the structure of the photosynthetic cell, which is most vulnerable to PAR [32,61]. After high light intensity and other stress factors, the processes of light absorption by photosynthetic pigments and the utilization of assimilation reactions were imbalanced. This rendered an increase in the risk of photodamage, occurring mainly in the result of ROS production (photooxidative damage). The reaction centers (RC) of PS I and PS II were the main sites of their generation. Light-induced formation of excited dimers ^3^P_680_* led to the production of ^1^O_2_ [32], although the formation of H_2_O_2_ in PS I RC has also been described [62]. Previously, it has been shown for *H. lacustris* that high photosynthetic activity in terms of PS II photochemical quantum yield led to the death of algal cultures [63]. Thus, it is vitally important to decrease the light absorption by PSA under HL. Excitation spectra of *C. rubescens* NAMSU R1 cells treated by HL or HL+UV-A were characterized by the decreasing of the fluorescence intensity in the violet, blue and blue–green region of the visible regions of the spectrum (Figure 3c). An analysis of the differential spectra (control minus treated) revealed the presence of a peak at 485 nm, corresponding to the absorbance of ketocarotenoids (Figure 3d). The comparison of absorbance and excitation spectra provided ground for the conclusion that carotenoids were involved in the optic shielding of PSA against HL.

Separation of the pigment extracts revealed that eight fractions corresponded with the carotenoids (Figure 2c). Two fractions with the highest R*_f_* (0.92 and 0.86) values were represented as most likely by β-carotene and α-carotene (Figure 2c; Table 1), because they were characterized by spectral details of these carotenoids. This was in accordance with previous data on the highest mobility of these carotenes, used in a separation system [41,51,56]. Other fractions were represented by xanthophylls (Figure 2c; Table 1). Four of them contained the pigments characterized by one maximum in the blue–green region of the spectrum, which is typical for ketocarotenoids and their esters with fatty acid [41,50,56]. The highest fraction of *C. rubescens* NAMSU R1 after treatment by HL or HL+UV-A was represented by astaxanthin, predominantly in the form of mono- and diesters (R*_f_* of 0.48 and 0.20–0.26, respectively) of fatty acids (Figure 2c; Table 1). This was determined by absorbance spectra and previous data on pigment distribution on the chromatogram [56]. Due to their higher hydrophobicity, astaxanthin diesters were characterized by higher mobility than monoesters. It was difficult to determine the exact R*f* value of the astaxanthin monoester fraction, due to large size of the spot that corresponded with it (Figure 2c). It could reflect high diversity of fatty acid residues of the esters. Minor fractions of astaxanthin biosynthesis intermediates (canthaxanthin, R*f* = 0.37, and echinenone, R*f* = 0.71) also presented in the chloroform extracts (Table 1). Minor fractions of carotenoids with the lowest mobility, i.e., high polarity, most likely, were represented by free astaxanthin and primary xanthophylls (Figure 2c, Table 1). It was difficult to obtain fractions of purified carotenoids in this case, because they were not well-separated. Nevertheless, we provided absorbance spectra of their fractions (Figure 2c). Based on the spectra, they contained chlorophyll impurities. Thus, the spectra were compensated to chlorophyll content for determination of the content of carotenoids. The pigment profile of *C. rubescens* NAMSU R1 after carotenoid synthesis induction was similar to that of another strain of *C. rubescens*, CCALA 475 [56]. In that case, most of the pigments were represented by astaxanthin esters, and the fraction of monoesters was the highest [56]. Similar data were obtained for other *Coelastrella*, e.g., *C. astaxanthina* Ki-4 [58], *C. aeroterrestrica* HELL-2 [51], *Coelastrella* sp. FGS-001 [59] and *C. oocystiformis* SAG-277/1 [60]. Particularly, Minyuk et al. [56] observed relatively high fractions of adonixanthin, echinenone and canthaxanthin in their extracts. Astaxanthin accumulation is a canonical mechanism of acclimation to adverse conditions in chlorophytes. Protection of cells of green microalgae against photodamage by the mechanism of optic shielding of PSA has previously been demonstrated for the carotenogenic chlorophyte *H. lacustris* [38,39,40]. In this microalga, astaxanthin accumulation leads to a decrease in the level of photoinhibition and cell viability [38] and affects the shape of chlorophyll excitation spectra [40]. In *H. lacustris,* astaxanthin esters represent up to 99% of total carotenoids [41,43,44,45,60], whereas some other microalgae accumulate significant fractions of other carotenoids, such as β-carotene, adonixanthin and adonirubin [51,56,60]. These pigments are intermediates of astaxanthin biosynthesis. A difference in carotenoid composition may be explained by a difference in the linkage between enzymes of ketocarotenoid synthesis pathways [64].

*H. lacustris* accumulates astaxanthin in cytoplasmic oil bodies (OB) [39,65]. These structures are subject to light-induced migration in the cell. This mechanism is mediated by actin microfilaments of the cytoskeleton [39]. Under high irradiation, oil globules with astaxanthin are located beyond the chloroplast. Such co-localization provides effective shielding of PSA against light. Another green microalga, *D. salina*, accumulates β-carotene in plastoglobuli [47,66].

**Figure 4:**
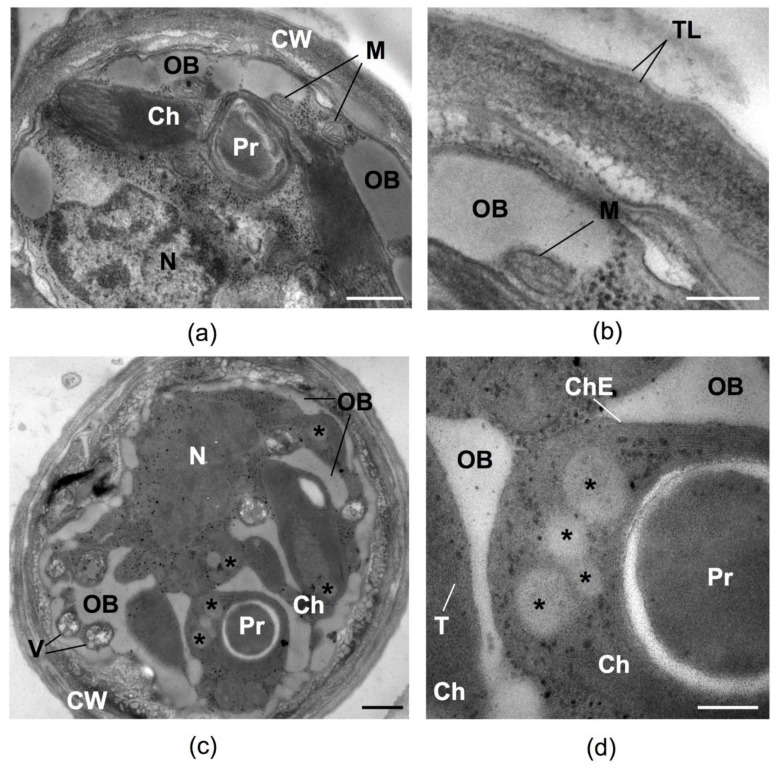
The ultrastructural features of the cells of *C. rubescens* NAMSU R1 before the treatment by HL (**a**,**b**) and after it (**c**,**d**); (**a**) common view of a cell before the stress; (**b**) enlarged fragment of the image (**a**) with a detailed view of the cell wall; (**c**) a common view of the cell after the stress; (**d**) enlarged fragment of the image (**c**) with special attention to plastoglobuli in the chloroplast. Ch—chloroplast; ChE—chloroplast envelope; M—mitochondrion; N—nucleus; OB—oil bodies; Pr—pyrenoid; TL—trilaminar layer. Asterisks point to plastoglobuli. Scale bars: (**a**,**c**)—0.5 µM, (**b**,**d**)—0.2 µM.

The cells of *C. rubescens* NAMSU R1 before HL or HL+UV-A treatment (Figure 4a,b) were characterized by a well-developed PSA and the presence of numerous mitochondria reflecting high metabolic activity. After irradiation by HL, the increase of the area occupied by lipid inclusions on the ultrathin cross-section was observed. These inclusions were localized at the cell periphery and tended to reduce the chloroplast compartment (Figure 4c,d). The cell wall became a more complex multi-layered spongiosum structure and a number of vacuoles increased. TEM observations revealed both types of lipid inclusions, cytoplasmic (Figure 4c,d) and plastidic (Figure 4d), in the cells after HL treatment. A small number of OB also presented in the cells before the HL treatment (Figure 4a,b). The shape of the cytoplasmic OB was irregular, whereas the plastoglobuli, located in chloroplast stroma, were round (Figure 4c,d). These inclusions also differed by their co-aggregation tendency: cytoplasmic OB (Figure 4c) merged, whereas plastoglobuli were located separately and did not tend to fuse (Figure 4d). These two types of lipid inclusions also differed in terms of electron opacity on TEM cross-sections: plastoglobuli had a higher electron density than OB. According to the commonly accepted paradigm, carotenes form *de novo* in chloroplasts of green algae, whereas their oxygenation—resulting in ketocarotenoid formation—takes place in the cytoplasm [37,42,67,68]. We speculated that carotenes were deposited in the plastoglobuli of *C. rubescens* NAMSU R1, whereas ketocarotenoids accumulated in the cytoplasmic OB. Despite the presence of a high percentage of β-carotene in the *C. rubescens* NAMSU R1 carotenoid profile (Table 1), and the presence of its features in the extract absorbance spectra (Figure 2b), no significant attenuation of chlorophyll fluorescence was observed in its absorption band. This might be explained by a higher fraction of xanthophylls, and a specific mutual localization of cytoplasmic globules and photosynthetic membranes, providing effective shielding of the PSA, which was not the case for plastoglobuli.

### 2.3. Sunscreen Effect in the UV Range

**Figure 5:**
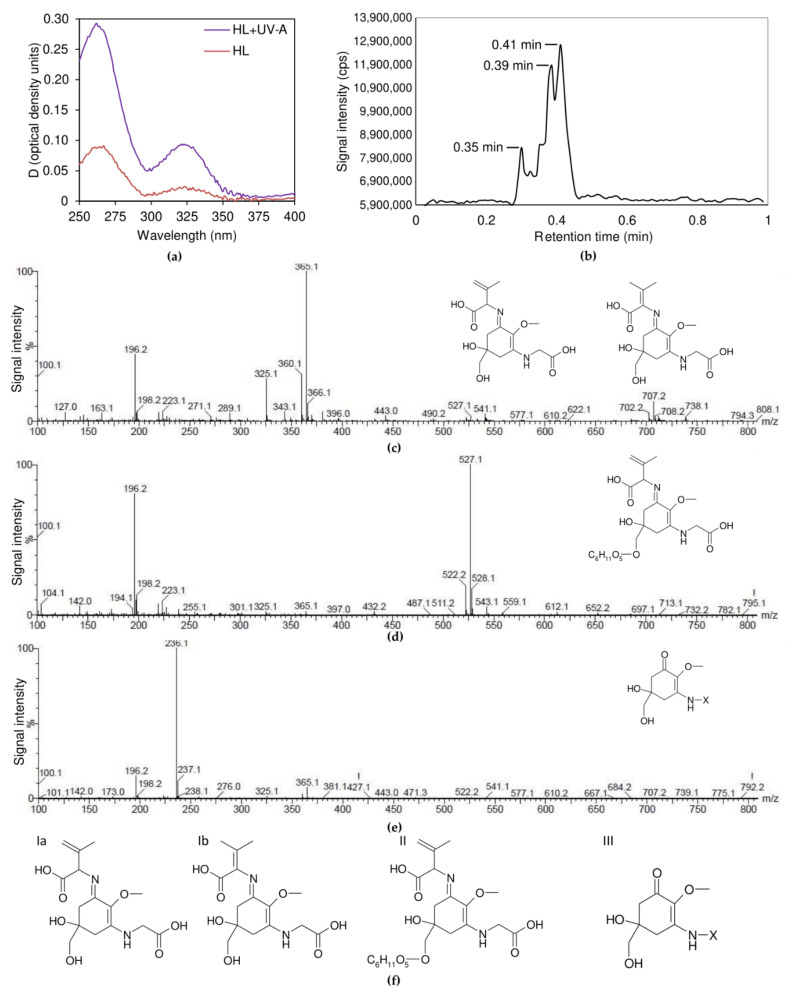
Analysis of mycosporine-like amino acids (MAA) from *Coelastrella rubescens* NAMSU R1 cells; (**a**) absorbance spectra of the water–methanol extracts of *C. rubescens* NAMSU R1 after treatment by HL and HL+UV-A registered against the control (*C. rubescens* NAMSU R1 cells treated by LL); (**b**) UPLC-separation of water–methanol extracts of *C. rubescens* NAMSU R1 cells treated by HL+UV-A; mass-spectra of UPLC fraction (**c**) at the retention time of 0.39 min; (**d**) at the retention time of 0.35 min; (**e**) at the retention time of 0.41 min (Y-axis is the ion flow intensity as a per cent of maximal value, X-axis is the *m*/*z* ratio), and (**f**) possible structures of MAA from each fraction of water–methanol extracts of *C. rubescens* NAMSU R1 cells obtained by UPLC, X is an unknown radical. cps—counts per second; HL—cells and extracts of the cells after HL treatment; HL+UV-A—cells and extracts of the cells after HL and UV-A treatment.

The absorbance spectra of the water–methanol extracts of *C. rubescens* NAMSU R1 cells treated by HL or HL+UV-A (Figure 5a) were characterized by two bands in the UV region, compared with the control extracts: the maximums were at 260 nm and 324 nm. In other words, the water–methanol extracts of the *C. rubescens* NAMSU R1 cells treated by HL+UV-A and HL showed enhancement of the absorption in the UV-range, compared with the control. This increase was more pronounced in the case of HL+UV-A, than HL only (Figure 5a). We hypothesized, based on the shape of the spectrum in the UV-A range, and based on the notion that the rise of the absorption was HL- and UV-A-inducible, that it corresponded with the MAA accumulation. No changes of the absorbance in the visible range of the spectrum were detected in the water–methanol extracts, compared with the control (Supplementary File S1). According to Karsten et al. [19], different lineages of green microalgae are characterized by different MAA composition, in terms of absorption characteristics: there are MAA with absorbance maximum at 322 and 324 nm. Notably, 324 nm-MAA have been reported in some Trebouxiophyceae, e.g., *Prasiola* spp. (Prasiolaceae), *Watanabea* spp. (Trebouxiaceae), *Pabia signiensis* (Trebouxiaceae), *Stichococcus* spp. (Stichococcaceae) and *Chlorella luteoviridis* (Chlorellaceae) [2,19]. The microalga *Lobosphaera incisa* (Trebouxiaceae) accumulates 322 nm-MAA [2]. The authors suggested that members of Ulvophyceae and Chlorophyceae did not accumulate in these compounds [2,19]. In addition, 324 nm-MAA also have been found in the charophyte *Klebsormidium* spp. (Klebsormidiaceae) [9,21,25].

The results from the separation of the water–methanol extracts from *C. rubescens* NAMSU R1 by ultra-performance liquid chromatography (UPLC) showed that they were characterized by the presence of highly hydrophilic components, which eluted by water with a minimal fraction of organic compounds. At least three components at the retention times (R*t*) of 0.30, 0.39 and 0.41 min were revealed in the extracts (Figure 5b). This was in accordance with existing data on MAA. Due to high hydrophilicity, MAA are eluted from reverse-phase columns in UPLC experiments by CH_3_CN or H_2_O solvents near the retention volume of non-sorbing components [2,16,19,69].

Nearly 30 types of MAA have been identified [14]. The chemical nature of MAA from green algae is relatively poorly studied [4]. Often, they are distinguished based on absorbance maximum and chromatographic retention times, R*t*, only [2,4,5,9,15,16,17,19,20,25]. Structures of some known molecules are presented, e.g., in [12,18,20,22], but most of them are characterized by the maximum of 330 nm or higher (such as palythene, palythinol, shinorine); the maximum of palythine, mycosporine-glycine and gadusol is at 320, 310 and 294 nm, respectively [2,9,12,13,14,15,16,35]. Two recently reported 324 nm-absorbing MAA named klebsormidin A (mycosporine-[glycosyl serine]) and klebsormidin B (mycosporine-serine) were purified by UPLC; their structure was resolved by 2D ^1^H/^13^C-NMR [21].

To reveal the chemical nature of MAA from *C. rubescens* NAMSU R1, treated by HL and UV-A, we studied them by UPLC, coupled to electrospray ionization (ESI) and quadrupole time-of-flight (TOF) mass spectrometry (MS), UPLC-ESI-TOF-MS (or briefly, UPLC-MS). The protonated molecules, [M+H]^+^, of most natural compounds in the ESI-MS undergo fragmentation in an ionization source. This results in the formation of characteristic fragment ions [70], making it possible to use obtained mass spectra for a viable primary identification of compounds in the mixture [70,71]. The value *m*/z = 343.1 of the protonated molecule [M+H]^+^ in the fraction with R*t* = 0.39 min (Table 2, Figure 5c) corresponded with the molecule M, with the mass of 342 Da. This is supported by the presence of the signal of additional cations, [M+NH_4_]^+^, [M+Na]^+^, and [M+K]^+^ (Table 2). Moreover, the signals of cluster ions [*n*M+H]^+^, [*n*M+NH_4_]^+^, [*n*M+Na]^+^, and [*n*M+K]^+^, where *n* = 2–5, were presented in the spectrum (Figure 5c, Table 2). The spectrum also contained characteristic fragment ions (Table 2). Comparison of the obtained spectrum with published data [17,21,69,72,73,74] suggested the fraction with R*t* = 0.39 min was represented by a aminocycloheximine-type MAA, namely, mycosporine-glycine:valine, with an additional double bond. Since MAA with an additional double bond in the cyclohexene ring had not been reported [4,9,13,17,24], an additional unsaturated bond had to be localized in the valine radical. Similarly, an analysis of the mass spectrum of the fraction with R*t* = 0.35 min (Figure 5d) showed the presence of the protonated ion [M+H]^+^ with *m*/*z* = 505.1 (Table 2). Its molecular mass was 162 Da higher than in the previous case, which corresponded to the mass of dehydrated hexose [70,71]. Thus, it might be represented by glycosylated form of the compound from the fraction with R*t* = 0.39 min. This was also supported by the fact of its higher hydrophilicity than in that fraction. The signal at the *m/z* = 432 was presented in the mass spectrum of the compound from the fraction with R*t* = 0.35 min (Table 2, Figure 5d), which could be identified as the glycine fragment of the ion [M+H]^+^. This meant that the hydroxyl group at the 5th C atom of cyclohexenimine ring might be glycosylated. Glycosylation in this position is commonly found in MAA from fungi and algae [15,18,23,75]. The mass spectrum of the fraction with R*t* = 0.41 min (Figure 5e) did not contain enough information for identification of the compound in this fraction. The presence of the characteristic ion with *m*/*z* = 236 indicated that this compound related to the oxocyclohexene type MAA [17]. It was impossible to determine the structure of the radical at 3rd C bonded N atom.

We propose the structure of the MAA of the *C. rubescens* NAMSU R1, based on mass spectra analysis (Figure 5f). The fraction with R*t* = 0.39 min might be presented by mycosporine-glycine with either β- or γ-dehydrovaline radical (compounds Ia and Ib). Saturated mycosporine-glycine:valine has an absorption maximum at 335 nm in 80% methanol [20]. It was previously found in some marine invertebrates [18], vertebrates [20], and in the haptophyte *Phaeocystis* [4], but was absent in green algae. Appearance of one additional double bond in the β-position of valine radical increases the size of the system of the conjugated double bonds in the molecule. It had to lead to a batochromic shift of the absorption maximum. At the same time, the *C. rubescens* NAMSU R1 water–methanol extract exhibited the maximum at a shorter wavelength (324 nm, Figure 5a). Therefore, the structure of the compound from the fraction with R*t* = 0.39 was Ib (Figure 5f), i.e., mycosporine-glycine:γ-dehydrovaline. We propose the name coelastrin A for this compound. A hypsochromic shift of the absorption maximum of *C. rubescens* NAMSU R1, compared with the maximum of mycosporine-glycine:valine from [20], could be explained by the difference in the methanol-to-water ratio and pH [76]. Similarly, the compound from the fraction with R*t* = 0.35 min was (*7-O-*hexosyl)-mycosporine-glycine:γ-dehydrovaline (compound II, Figure 5f). We propose the name coelastrin B for this. Unidentified oxocyclohexene type MAA with unknown radical (compound III, Figure 5f) was the compound from the fraction with R*t* = 0.41 min.

Collectively, we found two MAA with a determined chemical formula in *C. rubescens* (coelastrin A and coelastrin B) that had not previously been reported [15,17,18,19,20,21,69,72,73,74]. Characteristic ions (including cluster and fragment ions) in the mass spectra for their identification were found, which can be used on MAA identification by ESI-MS. It should be noted, however, that justification of found structures should be done in further research, e.g., by NMR analysis.

From an ecological point of view, MAA are an adaptation to a harmful effect of UV irradiation and other stress factors, such as osmotic or drought stress. In the case of organisms inhabiting the deep of seas and oceans, the task of photoprotection is partially solved, due to shielding beneath the surface water layers. By contrast, phototrophs in the upper water layers with high irradiation (photic zone), as well as aeroterrestrial species, should have more effective photoprotective mechanisms [2,4]. Indeed, MAA content in terrestrial microorganisms and microorganisms from upper layers of the sea is higher than in the depths [4]. Aeroterrestrial environments are characterized by harsher conditions (particularly, desiccation and high insolation) compared to water [2,3,17]. Thus *C. rubescens* might be an interesting model object for the studying of adverse effects on phototrophs. The UV-protective role of MAA has been shown in many studies previously [9,13,14]. These compounds are characterized by a high extinction coefficient and chemical stability [4,9,13,17,24], which make them excellent sunscreens [9,14,15,16,17,24]. Moreover, they are powerful antioxidants [13,14,16,24]; *C. rubescens* are known as a UV-tolerant alga [5]. At the same time, according to previous data, UV exposure has not been accompanied by MAA accumulation in some other chlorophycean microalgae [5]. We have, however, shown their presence in *C. rubescens* NAMSU R1.

The increase in the absorbance of the characteristic band of MAA in *C. rubescens* NAMSU R1 cells after HL and HL+UV-A treatment indicated that their synthesis in the microalga was HL- and UV-A-inducible. It could also be concluded that visible light and UV-A had a synergetic effect in this microalga, because a higher absorbance increase was observed in the case of HL+UV-A treatment, rather than in the case of HL only. This was in accordance with previous observations, that both high light and UV-A induce MAA accumulation [14]. There is no still clear answer as to whether UV-B promotes MAA synthesis [4,14,24]. For example, its accumulation is stimulated by both UV-A and UV-B in the rhodophyte *Agarophyton tenuistipitatum* [14], in the cyanobacterium *Aphanothece* [24] and in some chlorophytes, e.g., *Chlorella*, *Stichococcus*, and *Pseudococcomyxa* [2,5]. At the same time, another red alga, *Porphyra columbina* shows a decrease in MAA levels under HL+UV-A+UV-B, rather than under HL+UV-A only [14]. The same is true about the chlorophyte *Lobosphaera incisa* SAG 2007 [2]. Moreover, in *Mazzaella laminarioides*, treatment by both UV-A and UVA+UV-B leads to a reduction in MAA content [14]. The effect of UV-B on the synthesis of MAA in *C. rubescens* could be a matter of further works. MAA synthesis might be mediated by photoreceptors, e.g., phytochrome A, cryptochromes and phototropins. Their chromophore groups absorb UV-A radiation [10,11]. Nevertheless, the mechanisms of MAA synthesis regulation in microalgae are still poorly understood [24].

The absorbance spectra of *C. rubescens* NAMSU R1 cell suspensions that compensated to light scattering were characterized by a strong band at 250–350 nm (Figure 3a,b). A comparison of the spectra of cells (Figure 3a,b) and extracts (Figure 2b and Figure 5a) indicated that it could not be explained by only MAA presence. It seemed to be that the cells contained unextracted UV-absorbing compounds. Some studies were addressed to UV-absorbing properties of sporopollenin-like and algaenan-like polymers that accumulated in the cell walls of some species of microalgae [5,12,25,26]. Sporopollenin is a product of the phenylpropanoid pathway, whereas the acetate–malate pathway leads to algaenans [25]. It is a group of insoluble biopolymers characterized by high stability [25,77]. UV-absorption by sporopollenin-like compounds was previously shown for *C. rubescens* and some other green microalgae [5]. An evaluation of the *C. rubescens* NAMSU R1 cells by TEM (Figure 4b) revealed the presence of a specific three-layer (or trilaminar layer, TL) structure in the cell wall, characterized by the sizes of 19.9 ± 0.7 nm, corresponding with the literature data [78]. Such a layer has been previously observed for the strains of other species of the genus *Coelastrella* [55,59], which has corresponded with sporopollenin-like substances [25,55,59]. They are crucial for UV protection in some chlorophytes and charophytes [5,12,25,26]. An algaenan-like polymer was detected in the cell wall of *H. lacustris*, and also found in aeroterrestrial environments [79]. Sporopollenin-like and algaenan-like substances seem to be the key protectors of some microalgae against UV. Due to high stability, sporopollenin- or algaenan-like compounds provide a constant UV protection, whereas MAA synthesis is inducible [5,16,22,77].

The strong band with the maximum of 260–265 nm (Figure 5a) might correspond to aromatic residues of proteins. At the same time, increasing absorbance in this spectral range after HL+UV-A treatment might indicate the synthesis of phenolic compounds and flavonoids. These substances were identified previously in some close-related microalgae [3,9,80], but a more detailed analysis was required to determine their chemical nature.

Collectively, we have demonstrated the UV-inducible accumulation of MAA in Chlorophyceae for the first time. However, this was not the case of sporopollenin and algaenan-like substances. This could be explained by the absence of an increase in absorption in differential spectra, with and without the induction by HL or HL+UV-A (Figure 3b). This was in accordance with the previous concept of constitutive protection by these biopolymers [5,9].

### 2.4. Possible Photoprotective Mechanisms Additional to Sunscreen

Oxygenic phototrophic microorganisms (microalgae) exhibit a wide range of protective mechanisms against sunlight. They include ROS-neutralizing enzymes, cycles of carotenoid oxydation/de-epoxidation and DNA reparation [4,12,13,32]. However, the protection of cells by sunscreen has a serious advantage over enzymatic systems. Decreasing the amount of energy absorbed by photosynthetic pigment-protein complexes is a common strategy for aeroterrestrial phototrophs [9]. The first type of mechanisms is aimed at damage which has already occurred, whereas shielding behind sunscreen prevents photodamage [12]. However, we proposed that mechanisms preventing light absorption by PSA were additional to inducible sunscreen accumulation.

**Figure 6:**
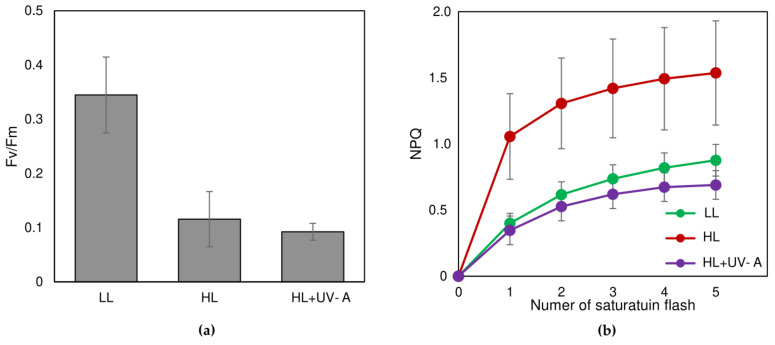
The parameters of the chlorophyll fluorescence induction curves of *Coelastrella rubescens* NAMSU R1 cells after 15 min dark acclimation. (**a**) Maximal photochemical quantum yield of PS II in the dark-acclimated state (Fv/Fm); (**b**) the Stern–Volmer parameter of non-photochemical quenching of the excited chlorophyll states (NPQ), as the function of the number of saturation light pulses during the illumination by actinic light. LL—cells treated by the low light; HL—cells treated by high light; HL+UV-A—cells treated by high light and UV-A. Average values from three replicates and standard deviations are shown.

The representative chlorophyll induction curves of *C. rubescens* NAMSU R1 are shown in Supplementary File S2. The cells of *C. rubescens* NAMSU R1 treated by HL or HL+UV-A demonstrated lower values of the PS II maximal photochemical quantum yield (Fv/Fm) than the cells treated by LL (Figure 6a). Such a decrease in Fv/Fm accompanying light-induced carotenoid accumulation has been reported for *H. lacustris* [38,40,81,82] and *D. salina* [83]. This could reflect the disassembly of PSA under stress conditions. Low Fv/Fm might be important to prevent a destructive photochemical reaction in the plastid ETC. Non-photochemical quenching of the excited chlorophyll states also plays a very important role in the photoprotection of PSA in higher plants and algae [32,50,84,85,86]. As a rule, this is up-regulated under stress conditions in order to shift the consumption of the absorbed light energy from chemical reactions to thermal dissipation and thus reduce the risk of photodamage. *C rubescens* NAMSU R1 cells treated by HL were characterized by the increase in NPQ, compared with LL-treated cells (Figure 6b). A sharp, short-term rise of NPQ has previously been well-documented for *H. lacustris*, cultured under the conditions of carotenoid synthesis induction (high light and/or depletion of the nitrogen source) [40,81,87]. It was a typical reaction of carotenogenic microalgae to stress factors. At the same time, buildup of the non-photochemical quenching was not observed after the treatment with HL+UV-A (Figure 6b). This could be explained by UV-induced damage of the proteins involved in non-photochemical quenching, or the significant contribution of slow-relaxing NPQ components, which did not relax during the time of dark acclimation. The latter could be reliable, considering the low values of Fv/Fm.

Absorption of PAR and UV by photosynthetic pigments causes the destruction of intracellular structures. The damaged chloroplast is a source of ROS and is not able to neutralize their high amount [32,86]. Thus, the turnover of non-functional plastids is vitally important to prevent the risk of photo-destruction. Autophagy is a process of self-regulation in the cell, consisting of selective isolation and the destroying of old, damaged, or abnormal substances and organelles in vacuoles. Chlorophagy, a complete degradation of damaged chloroplasts in vacuoles, is recognized as a special type of autophagy [88]. It has been described for a range of oxygenic photoautotrophic organisms, e.g., higher plants [88] and microalgae [89]. However, this is not the case of single-chloroplast microalgae, which cannot utilize whole chloroplast and perform the utilization of its fragments [84].

**Figure 7:**
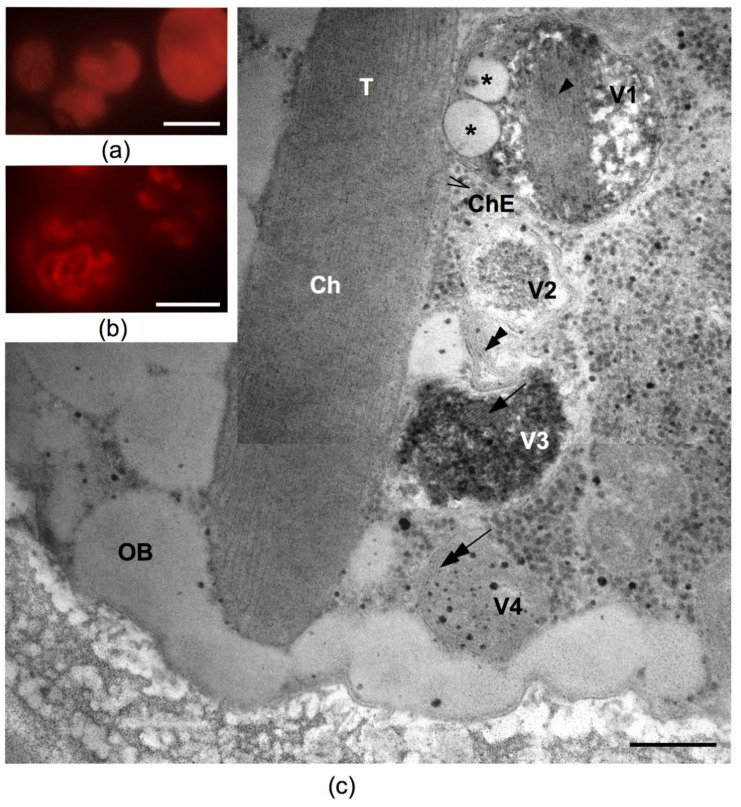
Photosynthetic apparatus reduction in the cells of *Coelastrella rubescens* NAMSU R1: (**a**,**b**) chlorophyll autofluorescence in the cells before HL treatment (**a**) and after it (**b**); (**c**) the ultrastructural features of an autophagy-like process in the cell after the treatment by HL. The image was compiled by combining images of two cell fragments. Twisted membranes in the cytoplasm ((**a**) double arrowhead) and vacuoles of four types are detected. V1—containing plastoglobuli-like globules and thylakoid-like membranes ((**a**) single arrowhead); V2—containing a loose material without membranes; V3—containing areas of amorphous inclusions of variable electron density with striped non-membrane structures (a single arrow); V4—with twisted membranes ((**a**) double arrow). Ch—chloroplast; ChE—chloroplast envelope; OB—oil bodies; V—vacuoles; T—thylakoids. Asterisks point to plastoglobuli-like globules. Scale bars: 5 µm (**a**,**b**), 0.2 µm (**c**).

That mechanism is conditioned by the rearrangement of membrane structures in the cell PSA and could be considered an autophagy-like process. In that case, thylakoids and sometimes whole chloroplasts are degraded to decrease the ability of cells to absorb light energy, which decreases the risk of photooxidative damage under stress conditions [84,85,88]. Under fluorescent microscope a reduction in the chloroplast after HL treatment can be seen in comparison with the cells, before it was observed, due to chlorophyll autofluorescence analysis (Figure 7a,b). That process, resulting in chloroplast content degradation, could be also visualized by TEM. The twirling pattern of the chloroplast envelope were observed on the TEM images of the cells of *C. rubescens* NAMSU R1, after their irradiation. They were similar to the epichloroplast membrane structures (EMS), described by Gorelova et al. [84]. HL-treated cells were also characterized by the presence of four types of vacuoles (Figure 7c). The first type (V1) contained plastoglobuli-like inclusions and membranes. The thickness of these membranes was similar to that of thylakoid membranes: 6.3 ± 0.3 nm and 6.2 ± 0.2 nm, respectively. The tonoplast of such vacuoles was in close proximity with the chloroplast outer membrane. According to Gorelova et al. [84] the transporting of membranes and stroma (due to the plastoglobuli-like structures presence in the vacuole) to a vacuole is one of the autophagy manifestations. The second type (V2) contained a loose material without membranes. The areas of amorphous inclusions were observed in the third type-vacuoles (V3). The inclusions exhibited variable electron density: the electron-dense areas alternated with transparent stripes of equal width. The complex of three “dark-light-dark” stripes (as in a membrane) observed in these vacuoles did not correspond to a thylakoid membrane, due to a significant difference in their width: 4.1 ± 0.2 nm vs. 6.2 ± 0.2 nm, respectively. These structures were similar to the previously described polyphosphate inclusions [90]. The last type (V4) contained twisted membranes, also similar to the EMS. Among described types of vacuoles, V1 and V4 were probably connected with autophagosome-like vesicles, because the fusion of autophagosome-like vesicles and tonoplast was detected. Moreover, they contained autophagic bodies. It is considered that the degree of chloroplast content degradation is to be dictated by the power and a type of stress affecting the cell [84,91,92]. The literature indicates that the observed features of the *C. rubescens* cells pointed to the emergency evacuation of the thylakoid membranes, and the chloroplast envelope was caused by the harsh effects of irradiation. The matter formed after the disassembly of PSA and other chloroplast components, with the resulting autophagy most likely being intended to fill in part the pool of neutral lipids [93,94,95]. Fatty acid residues may become the part of triacylglycerols of lipid inclusions. Later, under conditions favorable for photosynthesis, they can be used to assemble PSA [84,93]. The described process is also a marker of a cell autophagy and was detected in the studied cells (Figure 4c,d and Figure 7c).

Being the main site of photodamage, photosynthetic membranes should be reduced under HL, in order to avoid the irreversible destruction of the cell. In carotenoid-accumulating microalgae, the disassembly of PSA plays an important role in photoprotection along with carotenoid accumulation. Indeed, in *H. lacustris*, the reduction in PSA is important for the viability of cells under light, after their freezing [63]. The inhibition of autophagy in *H. lacustris* leads to an increase in carotenoid accumulation. This indicates competition between these photoprotective mechanisms [96]. The same was shown in the carotenogenic chlorophyte *Chromochloris zofingiensis* [53]. At the same time, the coexistence of different mechanisms, such as inducible sunscreen accumulation, the presence of a UV-absorbing sporopollenin-like layer and autophagy enhancement, is directed to the decrease in the level of the light potentially being absorbed by PSA.

## 3. Materials and Methods

### 3.1. Strain Isolation and Identification

The strain NAMSU R1 was isolated from the dry reddish crust (Figure 1a) on the *Malus × domestica* bark in the Rastorguevo Village, Moskovskaya oblast, Russia (55.55 N 37.70 E), in July of 2020. Strain isolation and maintenance were performed, based on previously developed protocol for the isolation of green carotenogenic microalgae [51].

To estimate the algal biomass accumulation, the strain NAMSU R1 was cultivated in 500 mL of the BG-11 media [97] in two 1 L Erlenmeyer flasks under continuous illumination, at a surface incident irradiance of 40 μmol/m^2^/s at 24 °C at 85 r.p.m in the New Brunswick Innova 44 shaking incubator (Eppendorf, Hamburg, Germany).

Primary identification and detailed morphological characterization and ultrastructure were performed by the evaluation of the culture by light and electron microscopy.

A precise identification was based on the sequence of ITS1-5.8S rRNA-ITS2. DNA extraction, fragment amplification and sequencing were performed, as previously described [98]. Sequences for the analysis were taken from the phylogenetic studies by Chekanov et al. [51], Kawasaki et al. [54], Kaufnerová and Eliáš [57], Wang et al. [58], and Goecke et al. [59]. The sequence JQ082314 of *Pectinodesmus pectinatus* CCAP 276/40 (Scenedesmaceae) was taken as an outgroup. Data analysis for the study of phylogenetic relationships was performed in MEGA X [99]. Sequences were aligned by the Muscle iterative algorithm [100]. Phylogeny was inferred by the Maximum Likelihood (ML) algorithm [101]. The Kimura two-parameter DNA evolution model [102] with an assumption of Gamma-distribution of substitution rates was selected for the ML analysis, under the Bayesian Information Criterion. Initial tree was constructed by the neighbor-joining algorithm [103] and the heuristic search was performed by the subtree pruning-regrafting method. The robustness of the tree topology was assessed by the bootstrap method [104], with 1000 replicates.

### 3.2. Induction of Photoprotectants’ Synthesis

The following LEDs were used in the work: UV-A LED (spectral range of 380–415 nm, power of 2.9 W/m^2^) and a cold-white LED (emission spectrum is presented in Supplementary File S3) with the photon flux density of 50 μmol/m^2^/s (“low light”, LL), and of 150 μmol/m^2^/s (“high light”, HL). Photon flux density of the visible light was measured at the level of cell suspensions by a LI-COR LI-250A quantum meter, with a cosine-corrected sensor (LI-COR Inc., Lincoln, NE, USA).

Suspensions of *C. rubescens* NAMSU R1 were taken from the stationary growth phase (15 days). The cells were transferred to 50 mL of BG-11 medium and diluted to the final optical density at 660 nm (D_660_) of 0.51 units. The strain NAMSU R1 was cultured in the 250 mL T-75 TC-treated cell culture flasks (Eppendorf, Hamburg, Germany) at 25 °C for three weeks. Two experimental variants were considered: cells were illuminated by HL (HL-variant) and by HL and UV-A (HL+UV-A). The variant of the cells illuminated by LL only was used as a control. The experiments were performed in three replicates.

### 3.3. Microscopy

#### 3.3.1. Light Microscopy

Microalgal cultures were evaluated by bright-field and fluorescent microscopy. This was performed using a Leica DM2500 microscope (Leica Microsystems, Wetzlar, Germany), equipped with the attached Leica DFC 700T camera. Chlorophyll autofluorescence was excited by a UV-lamp HXP 120 of the same manufacturer, using the band-pass filter Y3 (565–610 nm). The fluorescence was excited by a UV-shutter.

#### 3.3.2. Electron Microscopy

For scanning electron microscopy (SEM) the algal cells were fixed in 2% (*v*/*v*) glutaraldehyde and dehydrated through the graded ethanol series. Then, they were transferred to anhydrous acetone and dried at the CO_2_ critical point in a dryer HCP-2 (Hitachi, Tokyo, Japan). The samples then were sputter-coated with gold-palladium in an IB Ion Coater (Eiko, Tokyo, Japan) and examined with a JSM-6380LA (JEOL, Tokyo, Japan) scanning electron microscope at an accelerating voltage of 15 kV.

For transmission electron microscopy (TEM), the samples were fixed and dehydrated following the standard protocol described in Gorelova et al. [105]: fixed in 2% (*v*/*v*) glutaraldehyde solution in 0.1 M sodium cacodylate buffer, at room temperature for 0.5 h, and then post-fixed for 4 h in 1% (*wt*/*v*) osmium tetroxide in the same buffer. The samples were embedded in the epoxy resin araldite (Sigma-Aldrich, St. Louis, MO, USA). Ultrathin cross-sections were made using an ultramicrotome Leica EM UC7 (Leica Microsystems, Wetzlar, Germany) and a diamond knife Ultra 45° (DiATOME, Nidau, Switzerland). They were placed on a formvar-coated electron microscopy grids and contrasted with lead citrate [106]. Cross-sections were examined on JEM-1011 or JEM-1400 (JEOL, Tokyo, Japan) electron microscopes. The subcellular structures were measured (from 20 cells) on the TEM micrographs of the cell ultrathin sections using a Fiji (ImageJ) v. 20200708-1553 software (NIH, Bethesda, MA, USA). The data are shown as an average and their standard errors.

### 3.4. Spectroscopy

#### 3.4.1. Absorbance Spectra of Cell Suspensions

Absorbance spectra were registered in the range of 250–800 nm on an Agilent Cary 300 (Agilent, Santa Clara, CA, USA) spectrophotometer with a 150 mm integrative sphere CA30I (Agilent, Santa Clara, CA, USA) in 1 cm quartz cuvettes. The measured optical density compensated for the interferences from the incomplete collection of scattered light, as previously described [107,108].

The absorbance corrected to the effect of light scattering, *A_λ_*, was calculated as:

(1) $$A_{\lambda }=D_{\lambda }^{f}-D_{NIR}^{f}\frac{D_{\lambda }^{f}-D_{\lambda }^{c}}{D_{NIR}^{f}-D_{NIR}^{c}},$$

where $D_{\lambda }^{f}$ and $D_{\lambda }^{c}$ are the optical densities at the wavelength *λ* of the sample placed at a certain distance apart from the integrative sphere and as close as possible to it, respectively, $D_{NIR}^{f}$ and $D_{NIR}^{c}$ are the optical densities in the NIR region (at 800 nm) registered for the sample at a certain distance to the integrative sphere and close to the integrative sphere, respectively.

#### 3.4.2. UV-VIS-Absorbance Spectra of Cell Extracts

Putative sunscreens were extracted from the cells according to Folch et al. [109]. Before extraction, the microalgal cells were centrifuged at 12,000× *g*. The supernatant was removed, the biomass was frozen at the temperature of N_2_ boiling, and then the cells were disrupted using a ceramic mortar and a pestle.

Both water–methanol (hydrophilic) and chloroform (hydrophobic) fractions were collected and analyzed. The spectra of the fractions were recorded in the range of 250–800 nm on an Agilent Cary 300 (Agilent, Santa Clara, CA, USA) spectrophotometer in 1 cm quartz cuvettes. For HL and HL+UV-A-treated cells, the spectra were registered against the water–methanol extracts of the LL-treated *C. rubescens* NAMSU R1 cells as a blank. The carotenoid content in the biomass was determined as described in [50].

#### 3.4.3. Excitation Spectra

The excitation spectra of the microalgal cells were registered on an Infinite m200 microplate reader (Tecan, Grödig, Austria). Cell suspensions (2 mL) were transferred to a 24-well polypropylene plate (Corning Costar, Corning, NY, USA). Fluorescence was excited by the light in the UV and visible range of the spectrum (250–700 nm, bandwidth 9 nm); emission was detected at 750 nm (bandwidth 20 nm)—the band of chlorophyll *a* fluorescence. To compensate for the effect of fluorescence reabsorption due to the high density of cell suspension, the spectra were registered in a series of dilutions. The dilution range characterized by the linear correlation of the dilution and fluorescence intensity was eventually selected for the analysis (Supplementary File S4).

### 3.5. Chromatography

#### 3.5.1. Thin Layer Chromatography

The chloroform fraction of cell extracts (see Section 3.4.2) was analyzed by thin layer chromatography on a silica gel plate, Sulifol (Kavalier, Prague, Czech Republic). A previously developed mobile phase was used [41]. Pigment fractions obtained after the separation were analyzed, as has previously been described [50]. The chromatographic mobility factor (the retardation factor, R*f*) was calculated for the pigment fractions obtained after separation.

#### 3.5.2. Ultra-Performance Liquid Chromatography—Mass Spectrometry

Analysis of the sunscreens from the hydrophilic (methanol–chloroform) fraction (see Section 3.4.2) was performed by UPLC-ESI-TOF-MS. The samples were incubated at 40 °C in a rotary evaporator to remove methanol, dissolved in 1 mL of deionized water prepared on a Simplicity UV water purification system (Millipore, Molsheim, France), and filtered using 0.45 μm CAMEO 17F (Sigma-Aldrich, St. Louis, MO, USA). The obtained samples were separated by an ACQUITY UPLC H-Class PLUS (Waters Corporation, Milford, MA, USA), equipped with hybrid TOF mass spectrometer Xevo G2-XS Tof (Waters Corporation, Milford, MA, USA). A column ACQUITY UPLC BEH C18, 50 × 2.1 mm, 1.7 μM, (Waters Corporation, Milford, MA, USA) was used. The samples were separated at the temperature of 40 °C and the volumetric flow rate of 0.4 mL/min. The optimal protocol for separation was selected (Supplementary File S5), which was as follows. The system of solvents was used: 0.1% (*v*/*v*) HCOOH in H_2_O (component A) and 0.1% HCOOH in CH_3_OH (component B). Elution profile: 1% (*v*/*v*) A in B (0–1 min), 1→5% (*v*/*v*) A in B (1–5 min). The MS analysis was done in positive-mode ESI MS: *m*/*z* in the range of 100–2000, the ion source temperature of 150 °C, the desolvatation temperature of 200 °C, the capillary voltage of 4.5 kV, the ESI voltage of 30 V, and the desalvation gas (N_2_) flow rate of 461 L/h. Data were analyzed in a MassLynx software (Waters Corporation, Milford, MA, USA).

### 3.6. The Analysis of Chlorophyll Fluorescence Induction

Stationary chlorophyll fluorescence induction curves for the assessment of non-photochemical quenching of the excited chlorophyll states were recorded in a quartz cell (2 mm pathlength) with Fluorpen FP100 s PAM-fluorimeter (Photon System Instruments, Drásov, Czech Republic) after 15 mins dark adaptation, according to previously reported protocol [110]. The following parameters were calculated at each saturation light pulse during acclimation to the actinic light, according to Lazár [86]: the Stern–Volmer NPQ parameter,

(2) $$\mathrm{NPQ}\;=\frac{\mathrm{Fm}-\mathrm{Fm}\prime }{\mathrm{Fm}\prime },$$

where Fm and Fm’ are maximal chlorophyll fluorescence intensity in the dark-acclimated and light-acclimated state, respectively. In addition, maximal PSII photochemical quantum yield in the dark-acclimated state [86] was calculated as:

(3) $$\mathrm{Fv}/\mathrm{Fm}\;=\frac{\mathrm{Fm}-\mathrm{Fo}}{\mathrm{Fm}},$$

where Fo is the minimal chlorophyll fluorescence intensity in the dark-acclimated state.

## 4. Conclusions

A sunscreen effect provided by secondary carotenoids and MAA in response to HL and UV-A was described using a new strain of aeroterrestic microalga *C. rubescens* NAMSU R1. A complex of protective mechanisms, including shielding by chemical substances, and autophagy acting through a partial chloroplast reduction, were shown.

## Figures and Tables

**Table 1:** Fractions of carotenoids obtained after separation of the extracts from the cells of *Coelastrella rubescens* NAMSU R1 after an induction of carotenoid synthesis by HL and UV-A. Values of the retardation factor (R*f*), putative pigment name and its mass fraction (of total carotenoid content) in the extract are provided.

R*f*	Pigment	Content (%-of Total Carotenoid)
0.92	β-carotene	7.65
0.86	α-carotene	0.62
0.71	Echinenone	1.96
0.48	astaxanthin diesters	27.99
0.37	Canthaxanthin	0.97
0.20–0.26	astaxanthin monoesters	35.32
0.07	free ketocarotenoids + photosynthetic xanthophylls ^1^	4.94
0.06	photosynthetic xanthophylls	20.55

^1^ Xanthophylls of photosynthetic apparatus: zeaxanthin, antheraxanthin, violaxanthin, neoxanthin and lutein.

**Table 2:** Main characteristics detected by ESI-MS in the water–methanol extract of *C. rubescens* NAMSU R1 cells in different fractions, obtained after separation by UPLC. The retention times of UPLC fractions and *m*/*z* ratios are shown. M—studied MAA molecule.

Retention Time, Min.	*m*/*z*					
	[M+H]^+^	[M+NH_4_]^+^	[M+Na]^+^	[M+K]^+^	Cluster Ion	Fragment Ions
0.35	505.1	522.1	527.1	543.1	[2M+Na]^+^ 1031.3 [2M+NH_4_]^+^ 1026.3	487 365 325 432
0.39	343.1	360.1	365.1	381.1	[2M+Na]^+^ 707.2 [2M+NH_4_]^+^ 702.2 [2M+H]^+^ 685.2 [3M+Na]^+^ 1049.3 [3M+NH_4_]^+^ 1044.3 [3M+H]^+^ 1027.3 [4M+Na]^+^ 1391.4 [4M+NH_4_]^+^ 1386.4 [4M+H]^+^ 1369.4 [5M+Na]^+^ 1733.6 [5M+NH_4_]^+^ 1728.6 [5M+H]^+^ 1711.6	325 307 289 271 297 279 281 275 328
0.41	236.1	-	258.1	-	-	-

## Data Availability

The sequence of the ITS1-5.8S rRNA-IS2 DNA fragment for *Coelastrella rubescens* NAMSU R1 is available in the NCBI GenBank database under an accession number MZ230619.1.

## Supplementary Materials

The following are available online at https://www.mdpi.com/article/10.3390/plants10122601/s1, Supplementary File S1: absorbance spectra of the water-methanol fraction of the *Coelastrella rubescens* NAMSU R1 extracts in the UV and visible range, Supplementary File S2: chlorophyll *a* fluorescens induction curves of *Coelastrella rubescens* NAMSU R1 cells, Supplementary File S3: emission spectrum of the light emitting diodes used in the work, Supplementary File S4: selection of an optimal dilution range of *Coelastrella rubescens* NAMSU R1 suspensions for recording chlorophyll excitation spectra, Supplementary File S5: selection of the elution parameters for the analysis of the water-methanol extracts of *Coelastrella rubescens* NAMSU R1 by UPLC-MS.

## References

[1] Karsten U., Rindi F. (2010). Ecophysiological performance of an urban strain of the aeroterrestrial green alga *Klebsormidium* sp. (Klebsormidiales, Klebsormidiophyceae). Eur. J. Phycol..

[2] Karsten U., Lembcke S., Schumann R. (2007). The effects of ultraviolet radiation on photosynthetic performance, growth and sunscreen compounds in aeroterrestrial biofilm algae isolated from building facades. Planta.

[3] Holzinger A., Karsten U. (2013). Desiccation stress and tolerance in green algae: Consequences for ultrastructure, physiological and molecular mechanisms. Front. Plant Sci..

[4] Sen S., Mallick N. (2021). Mycosporine-like amino acids: Algal metabolites shaping the safety and sustainability profiles of commercial sunscreens. Algal Res..

[5] Xiong F., Komenda J., Kopecký J., Nedbal L. (1997). Strategies of ultraviolet-B protection in microscopic algae. Physiol. Plant..

[6] Goldberg B., Klein W.H. (1977). Variations in the spectral distribution of daylight at various geographical locations on the earth’s surface. Sol. Energy.

[7] Blumthaler M., Ambach W., Ellinger R. (1997). Increase in solar UV radiation with altitude. J. Photochem. Photobiol. B Biol..

[8] Kotilainen T., Aphalo P.J., Brelsford C.C., Böök H., Devraj S., Heikkilä A., Hernández A., Kylling A.V., Lindfors T.M., Robson T.M. (2020). Patterns in the spectral composition of sunlight and biologically meaningful spectral photon ratios as affected by atmospheric factors. Agric. For. Meteorol..

[9] Karsten U., Holzinger A. (2014). Green algae in alpine biological soil crust communities: Acclimation strategies against ultraviolet radiation and dehydration. Biodivers. Conserv..

[10] Verdaguer D., Jansen M.A., Llorens L., Morales L.O., Neugart S. (2017). UV-A radiation effects on higher plants: Exploring the known unknown. Plant. Sci..

[11] Vanhaelewyn L., Van Der Straeten D., De Coninck B., Vandenbussche F. (2020). Ultraviolet radiation from a plant perspective: The plant-microorganism context. Front. Plant Sci..

[12] Cockell C.S., Knowland J. (1999). Ultraviolet radiation screening compounds. Biol. Rev..

[13] Milito A., Castellano I., Damiani E. (2021). From Sea to Skin: Is There a Future for Natural Photoprotectants?. Mar. Drugs.

[14] Vega J., Schneider G., Moreira B.R., Herrera C., Bonomi-Barufi J., Figueroa F.L. (2021). Mycosporine-Like Amino Acids from Red Macroalgae: UV-Photoprotectors with Potential Cosmeceutical Applications. Appl. Sci..

[15] Oren A., Gunde-Cimerman N. (2007). Mycosporines and mycosporine-like amino acids: UV protectants or multipurpose secondary metabolites?. FEMS Microbiol. Lett..

[16] Singh A., Čížková M., Bišová K., Vítová M. (2021). Exploring Mycosporine-Like Amino Acids (MAAs) as Safe and Natural Protective Agents against UV-Induced Skin Damage. Antioxidants.

[17] Geraldes V., Pinto E. (2021). Mycosporine-like Amino Acids (MAAs): Biology. Chemistry and Identification Features. Pharmaceuticals.

[18] Wada N., Sakamoto T., Matsugo S. (2015). Mycosporine-like amino acids and their derivatives as natural antioxidants. Antioxidants.

[19] Karsten U., Friedl T., Schumann R., Hoyer K., Lembcke S. (2005). Mycosporine-like amino acids and phylogenies in green algae: Prasiola and its relatives from the Trebouxiophyceae (Chlorophyta). J. Phycol..

[20] Karentz D., McEuen F.S., Land M.C., Dunlap W.C. (1991). Survey of mycosporine-like amino acid compounds in Antarctic marine organisms: Potential protection from ultraviolet exposure. Mar. Biol..

[21] Hartmann A., Glaser K., Holzinger A., Ganzera M., Karsten U. (2020). Klebsormidin A and B, two new UV-sunscreen compounds in green microalgal *Interfilum* and *Klebsormidium* species (Streptophyta) from terrestrial habitats. Front. Microbiol..

[22] Singh S.P., Kumari S., Rastogi R.P., Singh K.L., Sinha R.P. (2008). Mycosporine-like amino acids (MAAs): Chemical structure, biosynthesis and significance as UV-absorbing/screening compounds. J. Exp. Biol..

[23] Nazifi E., Wada N., Asano T., Nishiuchi T., Iwamuro Y., Chinaka S., Matsugo S., Sakamoto T. (2015). Characterization of the chemical diversity of glycosylated mycosporine-like amino acids in the terrestrial cyanobacterium Nostoc commune. J. Photochem. Photobiol. B Biol..

[24] Rosic N.N. (2019). Mycosporine-like amino acids: Making the foundation for organic personalized sunscreens. Mar. Drugs.

[25] Holzinger A., Pichrtová M. (2016). Abiotic stress tolerance of charophyte green algae: New challenges for omics techniques. Front. Plant Sci..

[26] Procházková L., Remias D., Bilger W., Křížková H., Řezanka T., Nedbalová L. (2020). Cysts of the snow alga *Chloromonas krienitzii *(Chlorophyceae) show increased tolerance to ultraviolet radiation and elevated visible light. Front. Plant Sci..

[27] Carletti G., Nervo G., Cattivelli L. (2014). Flavonoids and melanins: A common strategy across two kingdoms. Int. J. Biol. Sci..

[28] Solhaug K.A., Gauslaa Y., Nybakken L., Bilger W. (2003). UV-induction of sun-screening pigments in lichens. New Phytol..

[29] Rao D.N., LeBlanc F. (1965). A possible role of atranorin in the lichen thallus. Bryologist.

[30] McEvoy M., Solhaug K.A., Gauslaa Y. (2007). Solar radiation screening in usnic acid-containing cortices of the lichen Nephroma arcticum. Symbiosis.

[31] Gauslaa Y., Ustvedt E.M. (2003). Is parietin a UV-B or a blue-light screening pigment in the lichen *Xanthoria parietina*?. Photochem. Photobiol. Sci..

[32] Asada K. (2006). Production and scavenging of reactive oxygen species in chloroplasts and their functions. Plant. Physiol..

[33] Merzlyak M.N., Chivkunova O.B. (2000). Light-stress-induced pigment changes and evidence for anthocyanin photoprotection in apples. J. Photochem. Photobiol. B Biol..

[34] Solovchenko A. (2010). Screening pigments: General questions. Photoprotection in Plants.

[35] Fernandes Â., Figueiredo S., Finimundy T.C., Pinela J., Tzortzakis N., Ivanov M., Soković M., Ferreira I., Petropoulos S.A., Barros L. (2021). Chemical composition and bioactive properties of purple French bean (*Phaseolus vulgaris* L.) as affected by water deficit irrigation and biostimulants application. Sustainability.

[36] Merzlyak M., Solovchenko A., Pogosyan S. (2005). Optical properties of rhodoxanthin accumulated in *Aloe arborescens* Mill. leaves under high-light stress with special reference to its photoprotective function. Photochem. Photobiol. Sci..

[37] Solovchenko A., Neverov K. (2017). Carotenogenic response in photosynthetic organisms: A colorful story. Photosynth. Res..

[38] Fan L., Vonshak A., Zarka A., Boussiba S. (1998). Does astaxanthin protect *Haematococcus* against light damage?. Z. Nat. C.

[39] Peled E., Pick U., Zarka A., Shimoni E., Leu S., Boussiba S. (2012). Light-induced oil globule migration in *Haematococcus pluvialis* (Chlorophyceae). J. Phycol..

[40] Chekanov K., Schastnaya E., Neverov K., Leu S., Boussiba S., Zarka A., Solovchenko A. (2019). Non-photochemical quenching in the cells of the carotenogenic chlorophyte *Haematococcus lacustris* under favorable conditions and under stress. Biochim. Biophys. Acta Gen. Subj..

[41] Chekanov K., Lobakova E., Selyakh I., Semenova L., Sidorov R., Solovchenko A. (2014). Accumulation of astaxanthin by a new *Haematococcus pluvialis* strain BM1 from the White Sea coastal rocks (Russia). Mar. Drugs.

[42] Takaichi S. (2011). Carotenoids in algae: Distributions, biosyntheses and functions. Mar. Drugs.

[43] Boussiba S., Vonshak A. (1991). Astaxanthin accumulation in the green alga *Haematococcus pluvialis*. Plant Cell Physiol..

[44] Lemoine Y., Schoefs B. (2010). Secondary ketocarotenoid astaxanthin biosynthesis in algae: A multifunctional response to stress. Photosynth. Res..

[45] Boussiba S. (2000). Carotenogenesis in the green alga *Haematococcus pluvialis*: Cellular physiology and stress response. Physiol. Plant..

[46] Ben-Amotz A., Shaish A., Avron M. (1989). Mode of action of the massively accumulated β-carotene of *Dunaliella bardawil* in protecting the alga against damage by excess irradiation. Plant. Physiol..

[47] Ben-Amotz A., Avron M., Cresswell R.C., Rees T.A.V., Shah N. (1989). The biotechnology of mass culturing Dunaliella for products of commercial interest. Algal and Cyanobacterial Biotechnology.

[48] Czygan F.C. (1968). Sekundär-Carotinoide in Grünalgen. Arch. Mikrobiol..

[49] Minyuk G.S., Chelebieva E.S., Chubchikova I.N. (2014). Secondary carotenogenesis of the green microalga *Bracteacoccus minor* (Chodat) Petrova (*Chlorophyta*) in a two-stage culture. Int. J. Algae.

[50] Chekanov K., Litvinov D., Fedorenko T., Chivkunova O., Lobakova E. (2021). Combined Production of Astaxanthin and β-Carotene in a New Strain of the Microalga Bracteacoccus aggregatus BM5/15 (IPPAS C-2045) Cultivated in Photobioreactor. Biology.

[51] Chekanov K., Fedorenko T., Kublanovskaya A., Litvinov D., Lobakova E. (2020). Diversity of carotenogenic microalgae in the White Sea polar region. FEMS Microbiol. Ecol..

[52] Procházková L., Leya T., Křížková H., Nedbalová L. (2019). *Sanguina nivaloid*es and *Sanguina aurantia* gen. et spp. nov. (Chlorophyta): The taxonomy, phylogeny, biogeography and ecology of two newly recognised algae causing red and orange snow. FEMS Microbiol. Ecol..

[53] Zhang Z., Sun D., Cheng K.W., Chen F. (2018). Inhibition of autophagy modulates astaxanthin and total fatty acid biosynthesis in *Chlorella zofingiensis* under nitrogen starvation. Biores. Technol..

[54] Kawasaki S., Yoshida R., Ohkoshi K., Toyoshima H. (2020). *Coelastrella astaxanthina* sp. nov. (Sphaeropleales, Chlorophyceae), a novel microalga isolated from an asphalt surface in midsummer in Japan. Phycol. Res..

[55] Tschaikner A., Ingolić E., Stoyneva M.P., Gärtner G. (2007). Autosporulation in the soil alga *Coelastrella terrestris* (Chlorophyta, Scenedesmaceae, Scenedesmoideae). Phytol. Balc..

[56] Minyuk G., Chelebieva E., Chubchikova I., Dantsyuk N., Drobetskaya I., Sakhon E., Chekanov K., Solovchenko A. (2017). Stress-induced secondary carotenogenesis in *Coelastrella rubescens* (Scenedesmaceae, Chlorophyta), a producer of value-added keto-carotenoids. Algae.

[57] Kaufnerová V., Eliáš M. (2013). The demise of the genus *Scotiellopsis* Vinatzer (Chlorophyta). Nova Hedwig..

[58] Wang Q., Song H., Liu X., Liu B., Hu Z., Liu G. (2019). Morphology and molecular phylogeny of coccoid green algae *Coelastrella sensu* lato (Scenedesmaceae, Sphaeropeales), including the description of three new species and two new varieties. J. Phycol..

[59] Goecke F., Noda J., Paliocha M., Gislerød H.R. (2020). Revision of *Coelastrella* (Scenedesmaceae, Chlorophyta) and first register of this green coccoid microalga for continental Norway. World J. Microbiol. Biotechnol..

[60] Orosa M., Torres E., Fidalgo P., Abalde J. (2000). Production and analysis of secondary carotenoids in green algae. J. Appl. Phycol..

[61] Melis A. (1999). Photosystem-II damage and repair cycle in chloroplasts: What modulates the rate of photodamage in vivo?. Trends Plant. Sci..

[62] Mehler A.H. (1951). Studies on reactions of illuminated chloroplasts: I. Mechanism of the reduction of oxygen and other hill reagents. Arch. Biochem. Biophys..

[63] Chekanov K., Vasilieva S., Solovchenko A., Lobakova E. (2018). Reduction of photosynthetic apparatus plays a key role in survival of the microalga *Haematococcus pluvialis* (Chlorophyceae) at freezing temperatures. Photosynthetica.

[64] Kim J.E., Cheng K.M., Craft N.E., Hamberger B., Douglas C.J. (2010). Over-expression of *Arabidopsis thaliana* carotenoid hydroxylases individually and in combination with a β-carotene ketolase provides insight into in vivo functions. Phytochemistry.

[65] Damiani M.C., Leonardi P.I., Pieroni O.I., Cáceres E.J. (2006). Ultrastructure of the cyst wall of *Haematococcus pluvialis* (Chlorophyceae): Wall development and behaviour during cyst germination. Phycologia.

[66] Polle J.E., Roth R., Ben-Amotz A., Goodenough U. (2020). Ultrastructure of the green alga Dunaliella salina strain CCAP19/18 (Chlorophyta) as investigated by quick-freeze deep-etch electron microscopy. Algal Res..

[67] Sun Z., Cunningham F.X., Gantt E. (1998). Differential expression of two isopentenyl pyrophosphate isomerases and enhanced carotenoid accumulation in a unicellular chlorophyte. Proc. Natl. Acad. Sci. USA.

[68] Cunningham F.X., Gantt E. (1998). Genes and enzymes of carotenoid biosynthesis in plants. Annu. Rev. Plant Biol..

[69] Parailloux M., Godin S., Fernandes S., Lobinski R. (2020). Untargeted Analysis for Mycosporines and Mycosporine-Like Amino Acids by Hydrophilic Interaction Liquid Chromatography (HILIC)—Electrospray Orbitrap MS2/MS3. Antioxidants.

[70] Kochkin D.V., Galishev B.A., Glagoleva E.S., Titova M.V., Nosov A.M. (2017). Rare triterpene glycoside of ginseng (ginsenoside malonyl-Rg 1) detected in plant cell suspension culture of Panax japonicus var. repens. Russ. J. Plant Physiol..

[71] Kochkin D.V., Galishev B.A., Titova M.V., Popova E.V., Nosov A.M. (2020). Chromato-Mass-Spectrometric Identification of Glycosides of Phenylethylamides of Hydroxycinnamic Acids in a Suspension Cell Culture of Mandragora turcomanica. Russ. J. Plant Physiol..

[72] Geraldes V., de Medeiros L.S., Lima S.T., Alvarenga D.O., Gacesa R., Long P.F., Fiore M.F., Pinto E. (2020). Genetic and biochemical evidence for redundant pathways leading to mycosporine-like amino acid biosynthesis in the cyanobacterium Sphaerospermopsis torques-reginae ITEP-024. Algae.

[73] Cardozo K.H.M., Vessecchi R., Carvalho V.M., Pinto E., Gates P.J., Colepicolo P., Galembeck S.E., Lopes N.P. (2008). A theoretical and mass spectrometry study of the fragmentation of mycosporine-like amino acids. Int. J. Mass Spectrom..

[74] Cardozo K.H., Carvalho V.M., Pinto E., Colepicolo P. (2006). Fragmentation of mycosporine-like amino acids by hydrogen/deuterium exchange and electrospray ionisation tandem mass spectrometry. Rapid Commun. Mass Spectrom..

[75] D’Agostino P.M., Javalkote V.S., Mazmouz R., Pickford R., Puranik P.R., Neilan B.A. (2016). Comparative profiling and discovery of novel glycosylated mycosporine-like amino acids in two strains of the cyanobacterium Scytonema cf. crispum. Appl. Environ. Microbiol..

[76] Matsuyama K., Matsumoto J., Yamamoto S., Nagasaki K., Inoue Y., Nishijima M., Mori T. (2015). pH-independent charge resonance mechanism for UV protective functions of shinorine and related mycosporine-like amino acids. J. Phys. Chem. A.

[77] Burczyk J., Zych M., Ioannidis N.E., Kotzabasis K. (2014). Polyamines in cell walls of chlorococcalean microalgae. Zeitschrift für Naturforschung C.

[78] Atkinson J.A., Gunning B.E.S., John P.C.L. (1972). Sporopollenin in the cell wall of Chlorella and other algae: Ultrastructure, chemistry, and incorporation of 14C-acetate, studied in synchronous cultures. Planta.

[79] Montsant A., Zarka A., Boussiba S. (2021). Presence of a nonhydrolyzable biopolymer in the cell wall of vegetative cells and astaxanthin-rich cysts of *Haematococcus pluvialis* (Chlorophyceae). Mar. Biotechnol..

[80] Goiris K., Muylaert K., Voorspoels S., Noten B., De Paepe D., Baart G.J.E., De Cooman L. (2014). Detection of flavonoids in microalgae from different evolutionary lineages. J. Phycol..

[81] Chekanov K., Lukyanov A., Boussiba S., Aflalo C., Solovchenko A. (2016). Modulation of photosynthetic activity and photoprotection in *Haematococcus pluvialis* cells during their conversion into haematocysts and back. Photosynth. Res..

[82] Torzillo G., Goksan T., Faraloni C., Kopecky J., Masojídek J. (2003). Interplay between photochemical activities and pigment composition in an outdoor culture of *Haematococcus pluvialis* during the shift from the green to red stage. J. Appl. Phycol..

[83] Gu W., Li H., Zhao P., Yu R., Pan G., Gao S., Xie X., Huang A., He L., Wang G. (2014). Quantitative proteomic analysis of thylakoid from two microalgae (*Haematococcus pluvialis* and *Dunaliella salina*) reveals two different high light-responsive strategies. Sci. Rep..

[84] Gorelova O., Baulina O., Ismagulova T., Kokabi K., Lobakova E., Selyakh I., Semenova L., Chivkunova O., Karpova O., Scherbakov P. (2019). Stress-induced changes in the ultrastructure of the photosynthetic apparatus of green microalgae. Protoplasma.

[85] Solovchenko A., Baulina O., Ptushenko O., Gorelova O. (2019). Ultrastructural patterns of photoacclimation and photodamage to photosynthetic algae cell under environmental stress. Physiol. Plant..

[86] Lazár D. (2015). Parameters of photosynthetic energy partitioning. J. Plant Physiol..

[87] Fratamico A., Tocquin P., Franck F. (2016). The chlorophyll a fluorescence induction curve in the green microalga *Haematococcus pluvialis*: Further insight into the nature of the P–S–M fluctuation and its relationship with the “low-wave” phenomenon at steady-state. Photosynth. Res..

[88] Izumi M., Ishida H., Nakamura S., Hidema J. (2017). Entire photodamaged chloroplasts are transported to the central vacuole by autophagy. Plant Cell.

[89] Pérez-Pérez M.E., Crespo J.L. (2010). Autophagy in the model alga *Chlamydomonas reinhardtii*. Autophagy.

[90] Shebanova A., Ismagulova T., Solovchenko A., Baulina O., Lobakova E., Ivanova A., Moiseenko A., Shaitan K., Polshakov V., Nedbal L. (2017). Versatility of the green microalga cell vacuole function as revealed by analytical transmission electron microscopy. Protoplasma.

[91] Baulina O., Gorelova O., Solovchenko A., Chivkunova O., Semenova L., Selyakh I., Scherbakov P., Burakova O., Lobakova E. (2016). Diversity of the nitrogen starvation responses in subarctic *Desmodesmus* sp. (Chlorophyceae) strains isolated from symbioses with invertebrates. FEMS Microbiol. Ecol..

[92] Scherbakov P., Ismagulova T., Chernov T., Gorelova O., Selyakh I., Semenova L., Baulina O., Chivkunova O., Solovchenko A. (2018). A new subarctic strain of Tetradesmus obliquus. Part II: Comparative studies of CO2-stress tolerance. J. Appl. Phycol..

[93] Goodson C., Roth R., Wang Z.T., Goodenough U. (2011). Structural correlates of cytoplasmic and chloroplast lipid body synthesis in *Chlamydomonas reinhardtii* and stimulation of lipid body production with acetate boost. Eukaryot. Cell.

[94] Goncalves E.C., Johnson J.V., Rathinasabapathi B. (2013). Conversion of membrane lipid acyl groups to triacylglycerol and formation of lipid bodies upon nitrogen starvation in biofuel green algae *Chlorella* UTEX29. Planta.

[95] Kong D.X., Li Y.Q., Wang M.L., Bai M., Zou R., Tang H., Wu H. (2016). Effects of light intensity on leaf photosynthetic characteristics, chloroplast structure, and alkaloid content of *Mahonia bodinieri* (Gagnep.) Laferr. Acta Physiol. Plant..

[96] Wang X., Song Y., Liu B., Hang W., Li R., Cui H., Li R., Jia X. (2020). Enhancement of astaxanthin biosynthesis in *Haematococcus pluvialis* via inhibition of autophagy by 3-methyladenine under high light. Algal Res..

[97] Stanier R.Y., Kunisawa R., Mandel M.C.B.G., Cohen-Bazire G. (1971). Purification and properties of unicellular blue-green algae (order *Chroococcales*). Bacteriol. Rev..

[98] Ismagulova T., Chekanov K., Gorelova O., Baulina O., Semenova L., Selyakh I., Chivkunova O., Karpova O., Lobakova E., Solovchenko A. (2018). A new subarctic strain of Tetradesmus obliquus—Part I: Identification and fatty acid profiling. J. Appl. Phycol..

[99] Kumar S., Stecher G., Li M., Knyaz C., Tamura K. (2018). MEGA X: Molecular evolutionary genetics analysis across computing platforms. Mol. Biol. Evol..

[100] Edgar R.C. (2004). MUSCLE: A multiple sequence alignment method with reduced time and space complexity. BMC Bioinform..

[101] Aldrich J. (1997). RA Fisher and the making of maximum likelihood 1912–1922. Stat. Sci..

[102] Kimura M. (1980). A simple method for estimating evolutionary rates of base substitutions through comparative studies of nucleotide sequences. J. Mol. Evol..

[103] Saitou N., Nei M. (1987). The neighbor-joining method: A new method for reconstructing phylogenetic trees. Mol. Biol. Evol..

[104] Felsenstein J. (1985). Confidence limits on phylogenies: An approach using the bootstrap. Evolution.

[105] Gorelova O.A., Baulina O.I., Solovchenko A.E., Chekanov K.A., Chivkunova O.B., Fedorenko T.A., Lobakova E.S. (2015). Similarity and diversity of the *Desmodesmus* spp. microalgae isolated from associations with White Sea invertebrates. Protoplasma.

[106] Reynolds E.S. (1963). The use of lead citrate at high pH as an electron-opaque stain in electron microscopy. J. Cell Biol..

[107] Merzlyak M.N., Naqvi K.R. (2000). On recording the true absorption spectrum and the scattering spectrum of a turbid sample: Application to cell suspensions of the cyanobacterium *Anabaena variabilis*. J. Photochem. Photobiol. B Biol..

[108] Merzlyak M.N., Chivkunova O.B., Maslova I.P., Naqvi K.R., Solovchenko A.E., Klyachko-Gurvich G.L. (2008). Light absorption and scattering by cell suspensions of some cyanobacteria and microalgae. Russ. J. Plant. Physiol..

[109] Folch J., Lees M., Stanley G.S. (1957). A simple method for the isolation and purification of total lipides from animal tissues. J. Biol. Chem..

[110] Chekanov K., Lobakova E. (2021). Photosynthesis measurements on the upper and lower side of the thallus of the foliose lichen *Nephroma arcticum* (L.) Torss. Photosynth. Res..

