# Supplementary material for: Sunscreen Effect Exerted by Secondary Carotenoids and Mycosporine-like Amino Acids in the Aeroterrestrial Chlorophyte Coelastrella rubescens under High Light and UV-A Irradiation
Source: Plants (Basel). 2021 Nov 26;10(12):2601. doi: 10.3390/plants10122601 (PMC8704241; doi:10.3390/plants10122601)
Supplement: Supplementary file 1 [file plants-10-02601-s001.zip › Supplementary S1.pdf]

Supplementary S1. Absorbance spectra of the water-methanol fraction of the *Coelastrella rubescens* NAMSU R1 extracts in the UV and visible range

Water-methanol extracts of the *Coelastrella rubescens* NAMSU R1 cells were prepared according to Folch et al. [1]. Before extraction, the microalgal cells were centrifuged at 12,000 g. The supernatant was removed, the biomass was frozen at the temperature of N_2 boiling, and then the cells were disrupted using a ceramic mortar and a pestle.
The water-methanol (hydrophilic) fraction was collected and analyzed. The following LEDs were used in the work: UV-A LED (spectral range of 380-415 nm, power of 2.9 W/m^2) and cold-white LED with the photon flux density of 50 $\mu mol/m^2/s$ ("low light", LL), and of 150 $\mu mol/m^2/s$ ("high light", HL). The spectra of the fractions were recorded in the range of 250–800 nm on an Agilent Cary 300 (Agilent, USA) spectrophotometer in 1-cm quartz cuvettes. The spectra of HL (**Figure S1a**) and HL+UV-A (**Figure S1b**) treated *C. rubescens* NAMSU R1 cells were recorded against the LL-treated cell extracts as a blanc. There were absorbance band in the UV-range: with the maximum at 260 nm, which might be due to the presence of nucleic acids, and at 324 nm, which, most likely, corresponded to MAA. No increasing of absorbance of the water-methanol extracts was detected in the visible range comparing to the control.

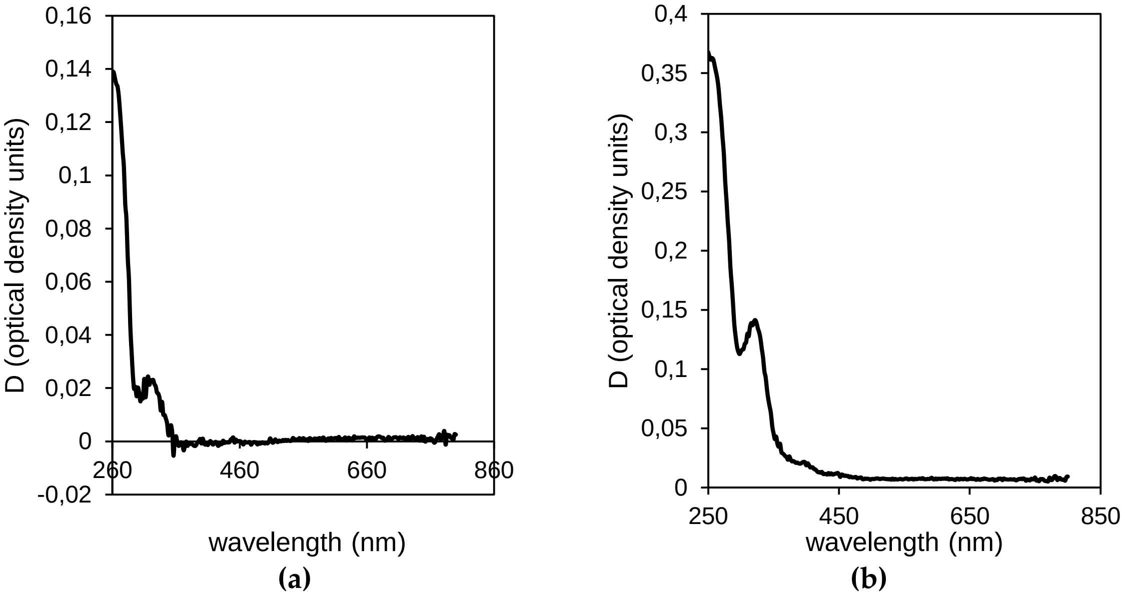

Figure S1. Absorbance spectra of the HL-treated (a) and HL+UV-A (b)-treated cells of Coelastrella rubescens NAMSU R1.

References

1. Folch, J.; Lees, M.; Stanley, G.S. A simple method for the isolation and purification of total lipides from animal tissues. *J Biol Chem* **1957**, *226*, 497–509.
