# Supplementary material for: Sunscreen Effect Exerted by Secondary Carotenoids and Mycosporine-like Amino Acids in the Aeroterrestrial Chlorophyte Coelastrella rubescens under High Light and UV-A Irradiation
Source: Plants (Basel). 2021 Nov 26;10(12):2601. doi: 10.3390/plants10122601 (PMC8704241; doi:10.3390/plants10122601)
Supplement: Supplementary file 1 [file plants-10-02601-s001.zip › Supplementary S2.pdf]

Supplementary S2. Chlorophyll *a* fluorescens induction curves of *Coelastrella rubescens* NAMSU R1 cells

Stationary chlorophyll fluorescence (CF) induction curves for assessment of non-photochemical quenching of the excited chlorophyll states were recorded in a quartz cell (2 mm pathlength) with Fluorpen FP100 s PAM-fluorimeter (Photon System Instruments, Czech Republic) after 15-min dark adaptation according to previously reported protocol [1]. Photon flux densities of the measuring and the saturating light were of 5 and of 3,000 µmol quanta/m²/s, respectively. CF was excited by a light-emitting diode (λ = 455 ± 5 nm) and detected within the range of 697–750 nm. Optical aperture diameter was of 5 mm. To induce regulatory non-photochemical quenching of the excited chlorophyll states, dark-adapted cells were exposed to actinic light of high photon flux density (λ = 455 ± 5 nm, 800 µmol quanta/m²/s), saturation pulse. was applied each 12 s during acclimation to actinic light for detection of maximal CF intensity in the light-adapted state (Fm'). Before actinic light exposure, Fo and Fm values were registered. Representative CF induction curves of *C. rubescens* NAMSU R1 treated by low light (50 µmol/m²/s) (Figure S2a), treated by high light (150 µmol/m²/s) (Figure S2b), c - treated by high light (150 µmol/m²/s) and UV-A (2.9 W/m²) (Figure S2c). There were a total of 5 flashes of saturation light during actinic light illumination. Representative Following parameters were calculated at each saturation light pulse during acclimation to the actinic light according to Lazár [2]: the Stern-Volmer NPQ parameter,

$$\mathrm{NPQ} = \frac{\mathrm{Fm} - \mathrm{Fm}'}{\mathrm{Fm}'},$$

the quantum yield of non-regulatory, φ(NO), and regulatory, φ(NPQ), dissipation,

$$\varphi(\mathrm{NO}) = \frac{\mathrm{Ft}}{\mathrm{Fm}'},$$

$$\varphi(\mathrm{NPQ}) = \varphi(\mathrm{NO}) \times NPQ,$$

where Ft is the stationary chlorophyll fluorescence intensity during actinic light acclimation, Fm and Fm′ are maximal chlorophyll fluorescence intensity un the dark-acclimated and light-acclimated state, respectively. In addition, maximal PSII photochemical quantum yield was calculated as [2]

$$\mathrm{Fv/Fm} = \frac{\mathrm{Fm} - \mathrm{Fo}}{\mathrm{Fm}},$$

where Fo is the minimal chlorophyll fluorescence intensity in the dark-acclimated state. Calculated parameters of quenching of the excited chlorophyll states are presented in the Table S2.

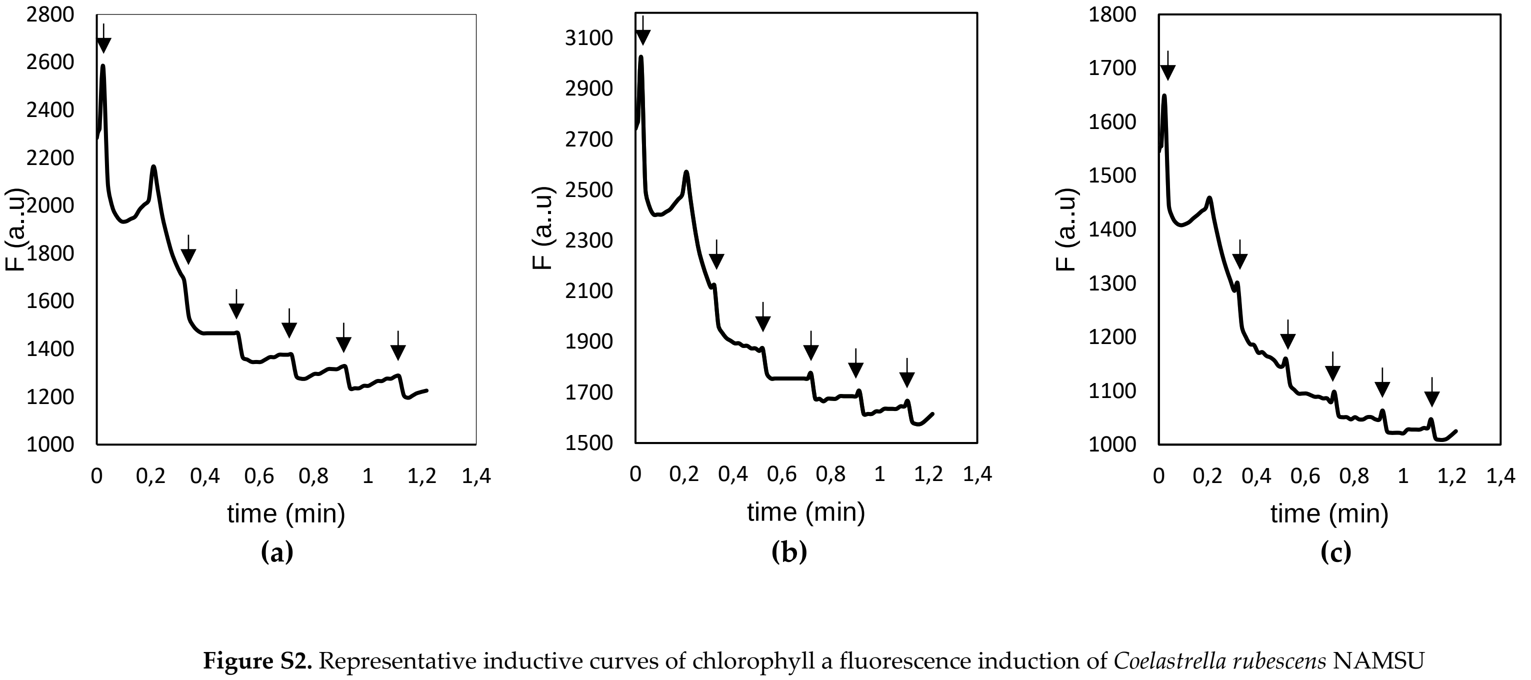

Figure S2. Representative inductive curves of chlorophyll a fluorescence induction of *Coelastrella rubescens* NAMSU R1 cells: a – treated by low light (50 µmol/m²/s), b – treated by high light (150 µmol/m²/s), c - treated by high light (150 µmol/m²/s) and UV-A (2.9 W/m²). a.u. – arbitrary units of chlorophyll fluorescence intensity. Arrows indicate saturation pulses.

Table S2. Calculated parameters of quenching of the excited chlorophyll states of the cells of *Coelastrella rubescens* NAMSU R1 at each saturation pulse. Average values from three replicates and their standard deviations are shown. φ(NO) – quantum yield of non-regulatory dissipation, φ(NPQ) – quantum yield of regulatory dissipation, NPQ – Stern-Volmer non-photochemical quenching parameter. * - zero saturatuin pulse applied for dark-adapted cells; in this case, φ(NO) was calculated as 1-Fv/Fm, , φ(NPQ) and were assumed as 0.

**# of**	**φ(NO)**			**φ(NPQ)**			**NPQ**		
**pulse**	**LL**	**HL**	**HL+UV**	**LL**	**HL**	**HL+UV**	**LL**	**HL**	**HL+UV**
0*	0,83±0.15	0,83±0.04	0,92±0.01	0	0	0	0	0	0
1	0,47±0.16	0,65±0.13	0,66±0.06	0,34±0.21	0,40±0.11	0,23±0.05	0,40±0.18	1,06±0.32	0,35±0.07
2	0,39±0.12	0,50±0.05	0,52±0.04	0,36±0.20	0,44±0.08	0,28±0.06	0,62±0.26	1,31±0.34	0,53±0.09
3	0,34±0.10	0,46±0.06	0,45±0.06	0,37±0.22	0,45±0.05	0,29±0.08	0,74±0.34	1,42±0.37	0,62±0.11
4	0,35±0.09	0,44±0.06	0,41±0.02	0,38±0.19	0,45±0.09	0,28±0.06	0,82±0.36	1,49±0.38	0,67±0.11
5	0,32±0.11	0,42±0.14	0,46±0.01	0,32±0.02	0,36±0.13	0,30±0.02	0,88±0.37	1,54±0.39	0,69±0.12

References

1. Chekanov, K..; Lobakova, E. Photosynthesis measurements on the upper and lower side of the thallus of the foliose lichen *Nephroma arcticum* (L.) Torss. *Photosynth Res* **2021**, *149*(3), 289-301.
2. Lazár, D. Parameters of photosynthetic energy partitioning. *J Plant Physiol* **2015**, *175*, 131-147.
