# Supplementary material for: Sunscreen Effect Exerted by Secondary Carotenoids and Mycosporine-like Amino Acids in the Aeroterrestrial Chlorophyte Coelastrella rubescens under High Light and UV-A Irradiation
Source: Plants (Basel). 2021 Nov 26;10(12):2601. doi: 10.3390/plants10122601 (PMC8704241; doi:10.3390/plants10122601)
Supplement: Supplementary file 1 [file plants-10-02601-s001.zip › Supplementary S3.pdf]

Supplementary S3. Emission spectrum of the light emitting diodes used in the work

Emission of LEDs for culturing of the cells of *Coelastrella rubescens* NAMSU R1 and sunscreens synthesis induction (Figure S1). The following LEDs were used in the work: UV-A LED (spectral range of 380-415 nm, power of 2.9 W/m²) and cold-white LED with the photon flux density of 50 µmol/m²/s ("low light", LL), and of 150 µmol/m²/s ("high light", HL) (Figure S3). Spectra were registered by an Ocean FX miniature spectrometer (Ocean Optics Inc., Great Britain) equipped with an integrative sphere ISP-80-8-R 80 mm in diameter of the same manufacturer.

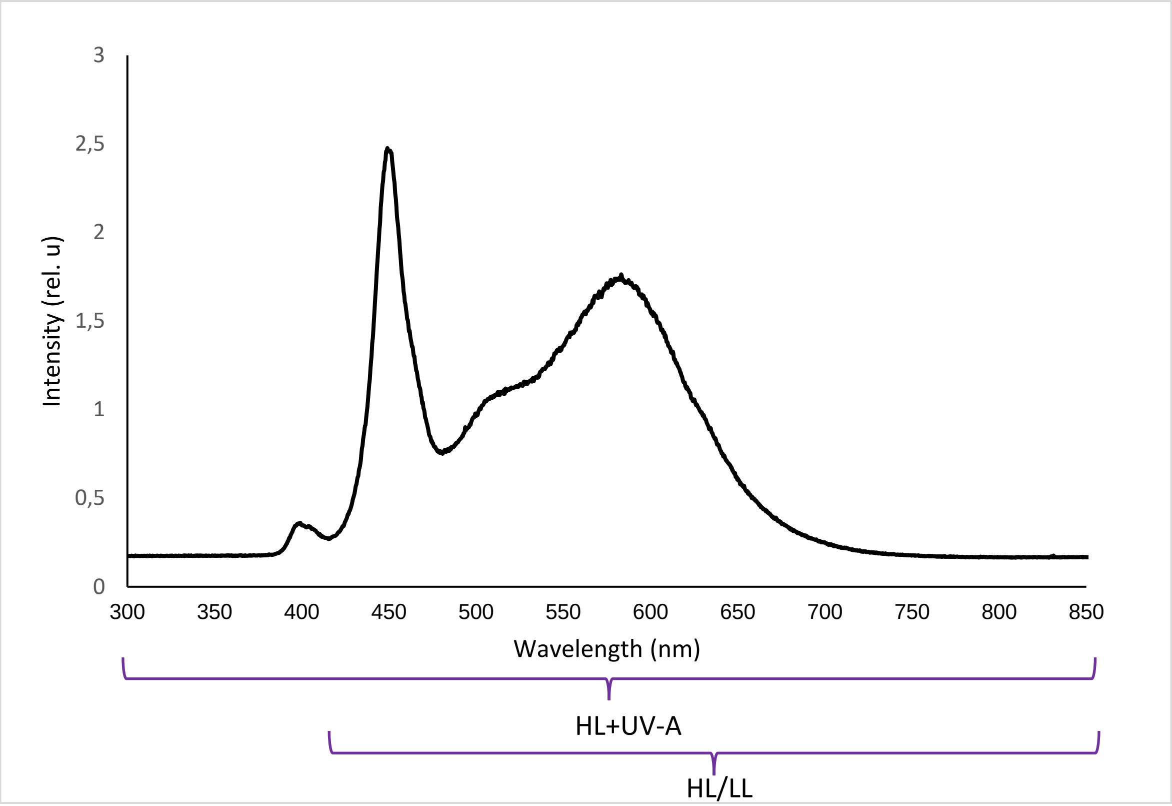

Figure S3. Emission spectrum of LEDs used for culturing of algal cells and sunscreen synthesis induction. HL, LL – part of the spectrum presented in the low light and high light. HL+UV-A combined high light and UV-A.
