# Supplementary material for: Sunscreen Effect Exerted by Secondary Carotenoids and Mycosporine-like Amino Acids in the Aeroterrestrial Chlorophyte Coelastrella rubescens under High Light and UV-A Irradiation
Source: Plants (Basel). 2021 Nov 26;10(12):2601. doi: 10.3390/plants10122601 (PMC8704241; doi:10.3390/plants10122601)
Supplement: Supplementary file 1 [file plants-10-02601-s001.zip › Supplementary S4.pdf]

Supplementary S4. Selection of an optimal dilution range of *Coelastrella rubescens* NAMSU R1 suspensions for recording chlorophyll excitation spectra

Fluorescence spectra of the *Coelastrella rubescens* NAMSU R1 cells were registered on an Infinite m200 microplate reader (Tecan, Austria). Cell suspensions (2 mL) were transferred to a 24-well polypropylene plate (Corning Costar, USA). Fluorescence was excited by the light at 480 nm; emission was detected at 660 nm (bandwidth 20 nm) - the band of chlorophyll *a* fluorescence. To compensate for the effect of fluorescence reabsorption due to high density of cell suspension, the spectra were registered in a series of dilutions, i.e. 0.166667 – 1.00000. Fluorescence intensity at 660 nm (laying in the zone of overlap between peaks of absorbance spectra and fluorescence emission spectra) as a function of dilution was analyzed (**Figure S4**). The spectra of *C. rubescens* treated by high light (HL, 150 μmol/m^2/s) and high light with UV-A (2.9 W/m^2, HL+UV-A). The cells treated by low light (50 μmol/m^2/s) were taken as a control. Based on the obtained data, the dilutions 1:5 and 1:4 were selected for the analysis.

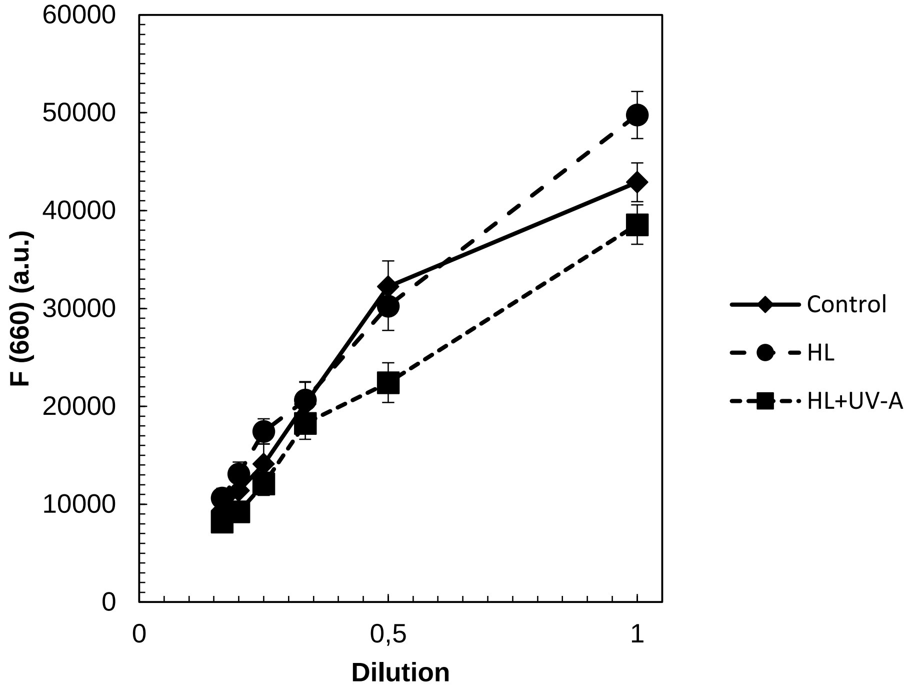

Figure S4. Fluorescence intensity at 660 nm in *Coelastrella rubescens* NAMSU R1 as a function of the dilution of cell suspensions. Medium values and standard deviations from three replicates are shown. Control – cells treated by low light, HL –cells treated by high light, HL+UV-A – cells treated by high light and UV-A.
