# Supplementary material for: Sunscreen Effect Exerted by Secondary Carotenoids and Mycosporine-like Amino Acids in the Aeroterrestrial Chlorophyte Coelastrella rubescens under High Light and UV-A Irradiation
Source: Plants (Basel). 2021 Nov 26;10(12):2601. doi: 10.3390/plants10122601 (PMC8704241; doi:10.3390/plants10122601)
Supplement: Supplementary file 1 [file plants-10-02601-s001.zip › Supplementary S5.pdf]

Supplementary S5. Selection of the elution parameters for the analysis of the water-methanol extracts of *Coelastrella rubescens* NAMSU R1 by UPLC-MS

The cells of *Coelastrella rubescens* NAMSU R1 incubated under high light (150 μmol/m^2/s) and UV-A (2.9 W/m^2) at 25 °C for three weeks were taken for the analysis because they were characterized by high absorbance in the UV range putatively corresponded to mycosporine-like amino acids (MAA). Water-methanol extracts were prepared according to Folch et al. [1]. For separation by ultra-performance liquid chromatography (UPLC), The samples were incubated at 40 ℃ in a rotary evaporator to remove methanol, dissolved in 1 mL of deionized water prepared on a Simplicity UV water purification system (Millipore, France) and filtered using 0.45 μm CAMEO 17F (Sigma-Aldrich, St. Louis, USA). A column ACQUITY UPLC BEH C18, 50×2,1 mm, 1,7 μm, (Waters, Massachusetts, USA) was used. The samples were separated at the temperature of 40 ℃ and the volumetric flow rate of 0.4 mL/min.

Four different separation protocols were studied (**Table S5**) they different by the composition of system of solvents for elution and elution profile. Due to high hidrophylity, MAA are eluted from reverse-phase columns by CH_3CN or H_2O solvents and near the retention volume of non-sorbing components [2]. Elution system consisted of two main components, A and B. The component A in all cases was presented by 0.1% (v/v) HCOOH in H_2O. The component B was either 0.1% (v/v) HCOOH in CH_3OH or 0.1% (v/v) HCOOH in CH_3CN.

Table S5. Different protocols of separation of *Coelastrella rubescens* NAMSU R1 water-methanol extracts by UPLC. The composition of the component B, its content and elution time are presented. * - protocol selected for the analysis by UPLC-MS.

Protocol	Component B	Content of component B, % (v/v)	Elution time, min
1	0.1% (v/v) HCOOH in CH_3CN	0→2	0-5
2	none	none	0-5
3	0.1% (v/v) HCOOH in CH_3OH	0→5	0-1
		5→10	1-5
4*	0.1% (v/v) HCOOH in CH_3OH	0→2	0-5

The extracts of *C. rubescens* were characterized by presence of highly hydrophilic components, which eluted by water with minimal fraction of organic compounds which is in accordance with existing data on MAA [2]. Only one pronounced peak was observed in the chromatogram obtained by the protocols 1 (**Figure S5a**) and 3 (**Figure S5b**). At the same time, at least three components were reveled in the chromatograms obtained by the protocols 2 (**Figure S5c**) and 4 (**Figure S5d**). Due to better separation of peacs the protocol 4 was selected to subsequent UHPLC-MS analysis.

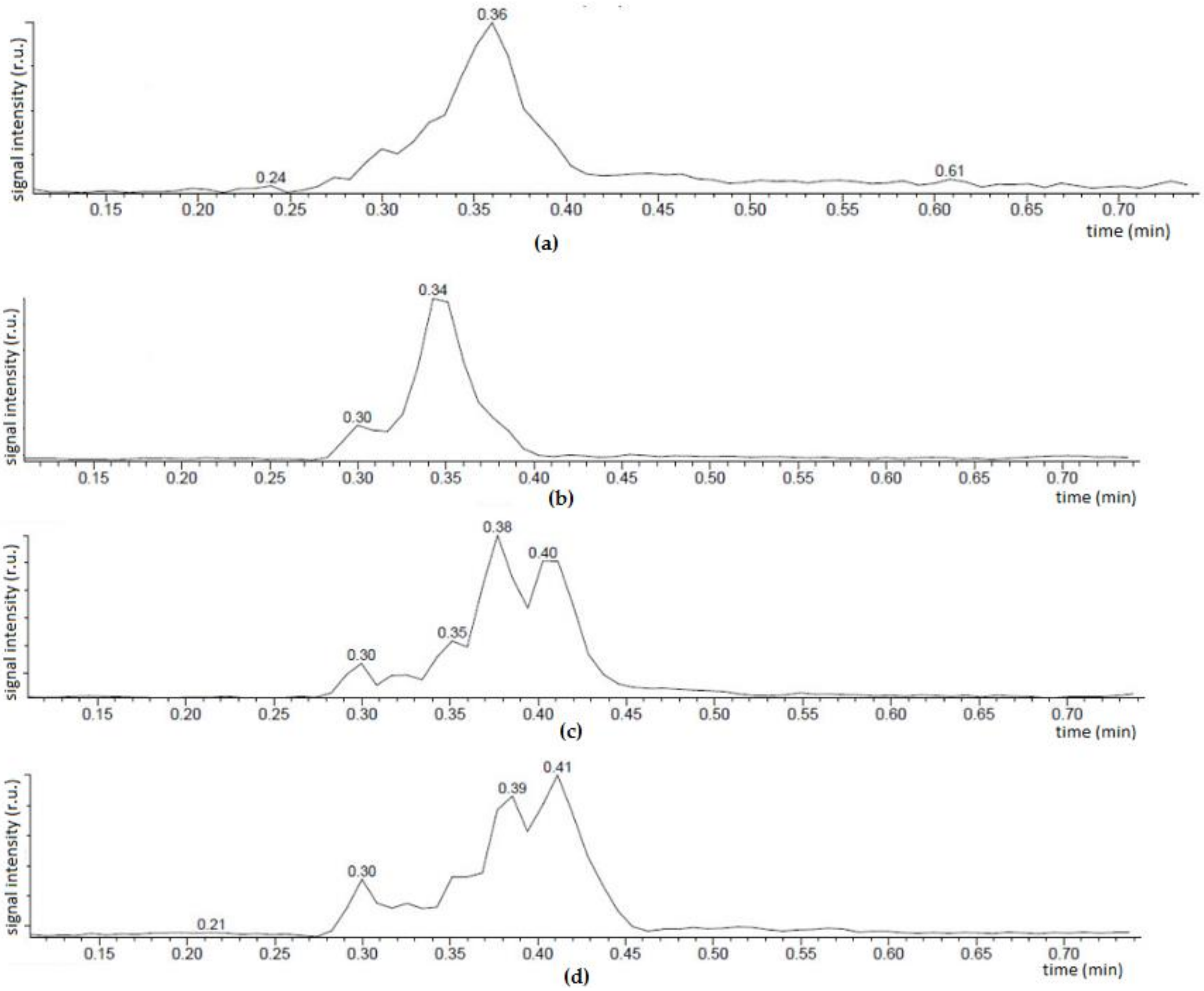

Figure S5. Chromatograms of the water-methanol extracts of *Coelastrella rubescens* NAMSU R1 treated by HL+UV-A obtained by UPLC protocols 1 (a), 3(b), 2 (c) and 4(d).

References

1. Folch, J.; Lees, M.; Stanley, G.S. A simple method for the isolation and purification of total lipides from animal tissues. *J Biol Chem* **1957**, *226*, 497–509.
2. Parailloux, M.; Godin, S.; Fernandes, S.; Lobinski, R. Untargeted Analysis for Mycosporines and Mycosporine-Like Amino Acids by Hydrophilic Interaction Liquid Chromatography (HILIC)—Electrospray Orbitrap MS^2/MS^3. *Antioxidants* **2020**, *9*(12), 1185.
